# Assessment of functional and structural changes of soil fungal and oomycete communities in holm oak declined *dehesas* through metabarcoding analysis

**DOI:** 10.1038/s41598-019-41804-y

**Published:** 2019-03-29

**Authors:** Francisco J. Ruiz Gómez, Rafael M. Navarro-Cerrillo, Alejandro Pérez-de-Luque, Wolfgang Oβwald, Andrea Vannini, Carmen Morales-Rodríguez

**Affiliations:** 1 Departamento de Ingeniería Forestal, Laboratorio de Ecofisiología de Sistemas Forestales ECSIFOR- ERSAF. Universidad de Córdoba. Campus de Rabanales, Crta. IV, km. 396, E-14071 Córdoba, Spain; 2 Área de Genómica y Biotecnología, IFAPA, Centro Alameda del Obispo, Avda. Menéndez Pidal s/n, Apdo, 3092, 14080 Córdoba, Spain; 3 Fachgebiet Pathologie der Waldbäume, Technische Universität München. Hans-Carl-von-Carlowitz-Platz 2, 85354 Freising, Germany; 4 Department for Innovation in Biological, Agro-food and Forest systems (DIBAF) –University of Tuscia, Via San Camillo de Lellis snc, Viterbo, 01100 Italy

## Abstract

Forest decline is nowadays a major challenge for ecosystem sustainability. *Dehesas*, which consists of savannah-like mediterranean ecosystems, are threatened by the holm oak decline in the south-west of Iberian Peninsula. *Phytophthora cinnamomi* is considered the main agent of holm oak root rot, but little is known about the relationship between diversity of soilborne microbial community and the decline syndrome of holm oak. It would be hypothesized that the changes in the structure and functionality of the soil microbiome might influence tree health status through changes in richness and diversity of beneficial organisms such as mycorrhizal species, or fungal plant pathogens such as *Fusarium* spp. or *Alternaria* spp. Total DNA of soil samples from declined oak dehesas was extracted and analyzed through metabarcoding techniques, to evaluate the specific composition and diversity of the fungal and oomycete communities and their relationship with the disease symptoms. The fungal community included a wide range of pathogens and abundance of ectomycorrhizal key taxa related with low defoliation degree. *Phytophthora cinnamomi* and *Pythium spiculum* did not appear among the most abundant oomycetes, nor were they related directly to defoliation levels. Moreover, a particular taxon belonging to the genus *Trichoderma* was strongly correlated with the scarcity of pathogenic *Phytophthora* spp. The diversity and composition of fungal and oomycete communities were related to the severity of the decline symptoms. The metabarcoding study of microbiome represents a powerful tool to develop biocontrol strategies for the management of the holm oak root rot.

## Introduction

Alien-invasive species and global change are nowadays major threats for ecosystems sustainability around the world. It is possible to find examples of different die-back syndrome of woody species devastating natural or semi natural ecosystem around the world related with these species^1^. In the last decades, different pathogenic *Phytophthora* spp. and *Pythium* spp. are emerging as important invasive pathogen species, considering the environmental losses they cause^2,3^. Some examples are *Phytophthora ramorum* Werres in Sudden Oak Death, affecting tanoak (*Notholithoarpus densiflorus* (Hook. & Arn.) Manos, Cannon & S.H.Oh) and several oak species in California^4^, *Phytophthora cinnamomi* Rands. in eucalyptus and Jarrah ecosystem in Australia^5^, or *Phytophthora cryptogea* Pethybr. & Laff*., Phytophthora cambivora* (Petri) Buisman and *P. cinnamomi* causing ink disease in chestnuts worldwide^6^.

Among the most relevant diseases caused on European woody species by *Phytophthora* spp., root rot has been identified since the 1990’s affecting oaks^7^. Sessile and pedunculated oaks (*Quercus petraea* (Matt.), Liebl. and *Quercus robur* L.) from North and Central Europe are threatened by several *Phytophtora* spp.^8^, and in the Mediterranean basin, the decline of holm oak (*Quercus ilex* L., subsp. *ballota* in Spain and Portugal, and subsp. *ilex* in central Italy) and cork oak (*Quercus suber* L.) due to root rot were identified since the early 1980’s, affecting *dehesa* rangelands in Spain and Portugal, and wide forest areas in France and Italy^9–11^.

*Dehesas* represent ecosystems with relevant ecological and socio-economic importance covering 23% of the forested area in Spain (around 14% of the total area), accounting for over 3 mill ha in the Iberian Peninsula^12^. In general, *dehesa* is a traditional land use in areas excluded from intensive agriculture, covering large extension of territory mostly characterized by a typical Mediterranean climate and soils usually shallow, acidic and deficient in nutrients^13^. This agroecosystem is mainly composed by open woodlands of *Quercus ilex* and *Q. suber*, with an annual grassland understory, presenting canopy covers ranging from 5 to 60%^14^. *Dehesa* ecosystem provides important socioeconomic benefits and many ecosystem services, such as shelter for a high degree of biodiversity or soil erosion prevention, being considered a first line barrier against desertification^15^.

Tree mortality of holm oak (*Quercus ilex* L.) associated with root rot is a serious threat to the sustainability of these ecosystems. *Phytophthora cinnamomi* Rands. is considered the main agent of this disease^16,17^, but the influence of other biotic and abiotic factors - such as the increasing frequency of extreme drought episodes and decreasing precipitation associated with global warming - is also recognized^18^. Research efforts on holm oak decline have been focused on the interaction between the host plants and the pathogen – *Q. ilex* and *P. cinnamomi*^19–21^, and on their relationships with environmental and human factors, including socio-economic and management strategies^22,23^. Management strategies for the control of disease spreading are often difficult to implement because most of the *dehesas* yield is based on cattle livestock or hunting game^11^. On the other hand, other treatments such as calcium soil amendments and phosphorous fertilization influenced tree health status but are unable to suppress the soil inoculum^24,25^. The application of sinigrin from Brassicaceae cultivars has been shown to be effective for pathogen suppression in controlled conditions experiments, but the biomass production necessary for an effective bio-amendment on the field is far greater than the productive ability of the *dehesa* soils^26^. However, the relationship between the plant community and oomycetes and the spatial patterns of *Pythium* spp. and *Phytophthora* spp. were shown to be relevant factors influencing the dynamics of declining Mediterranean forests^27^. Moreover, the implication of other *Phytophthora* spp. in the decline of *Q. ilex* and other *Quercus* spp. in the Iberian Peninsula is recognized^28–30^.

In addition, little is known about the role of the soil fungal community in the decline syndrome of holm oak, or about the relationship between the latter and the presence of pathogenic species belonging to oomycetes. Different authors have highlighted the importance of the interactions among communities of soil taxa, mainly focusing on bacterial and fungal communities^31,32^. The soil fungal community interacts with other communities of biota in different ways, acting as commensal, pathogenic, antagonistic or mutualistic organisms. In the case of the plant community, its interaction is mainly associated with beneficial species such as ectomycorrhizal (EcM) and mycorrhizal (AM) fungi and other soil saprobes which improve soil fertility and structure and enhance plant defenses and health status^33^. Ectomycorrhizal and endophytic species have been found to be related to changes in the tree health status of several broad-leafed species, including *Q. ilex*^22,34–36^. On the other hand, pathogenic relationships between fungal species and the plant community, including asymptomatic ones, are also related to disease expression in some cases^37^.

Oomycete communities are composed of a wide range of plant pathogens, some of which are host-specific and others with a wide range of hosts. Their lifestyle can be either biotrophic, necrotrophic and hemibiotrophic^38,39^. *Phytophthora* spp. communities have often been found to be the dominant in forest soils with a known history of *Phytophthora*-related diseases^40^, but little is known about the relationship between the oomycete community and soil fungal or bacterial communities. Sapkota and Nicolaisen^41^ published the first study of the soil community diversity, including the taxa of fungi and oomycetes, associated with cavity spot of carrot, but in the literature, there are no studies describing fungal diversity or the relationship between oomycetes and fungi in *dehesas* rangeland.

The isolation of living cultures was the traditional way to assess oomycete or fungal diversity in soils, but this approach seems to be insufficient due to the time and specialized scientific knowledge required for phenotypic species identification^42^, considering the potentially enormous number of different species – 1.5 to 3 million fungal species^43^ – in a high number of samples. In this sense, next-generation sequencing (NGS) techniques are an alternative methodology, able to detect the presence of a very high number of different taxa in a single sample: successful examples include pyrosequencing^40,44^ and second-generation techniques, such as metabarcoding based on Illumina de novo sequencing^41,45,46^. The ability of metabarcoding techniques to characterize high-complex soil microbiome systems has represented an extraordinary improvement on the study of the plants and associated microbiota interaction. These techniques also offer new opportunities for the deep assessment of microbiome ecology, given an important tool to optimize integrated control methods on management of plant diseases^47^.

The main objective of our work was to assess and describe the diversity and structure of fungal and oomycete communities in the holm oak *dehesas* rangeland ecosystems of Andalusia (southern Spain) affected by oak decline, using a metabarcoding approach, to analyse the correlations of structure and functionality of these communities between them, and with the defoliation level of *Q. ilex*. We hypothesize that the interaction between the microbiota diversity and functionality must be related with changes in tree health status, considering that fungi and oomycete are the main species involved in the holm oak decline due to root rot. To reach our main objective, the following specific objectives were addressed: i) to compare the fungal and oomycete diversity, and two defoliation levels in holm oak stands, in four different study zones; ii) to identify the predominant functional guilds of the fungal and oomycete communities and their relationships; and iii) to identify key taxa which might be related to holm oak decline. These results should contribute to a better understanding of the influence of soil community associations on tree health status and to the identification of important fungal and oomycete taxa related to holm oak decline in *dehesas* ecosystems.

## Results

### OTUs clustering and taxonomy

**Figure 1:**
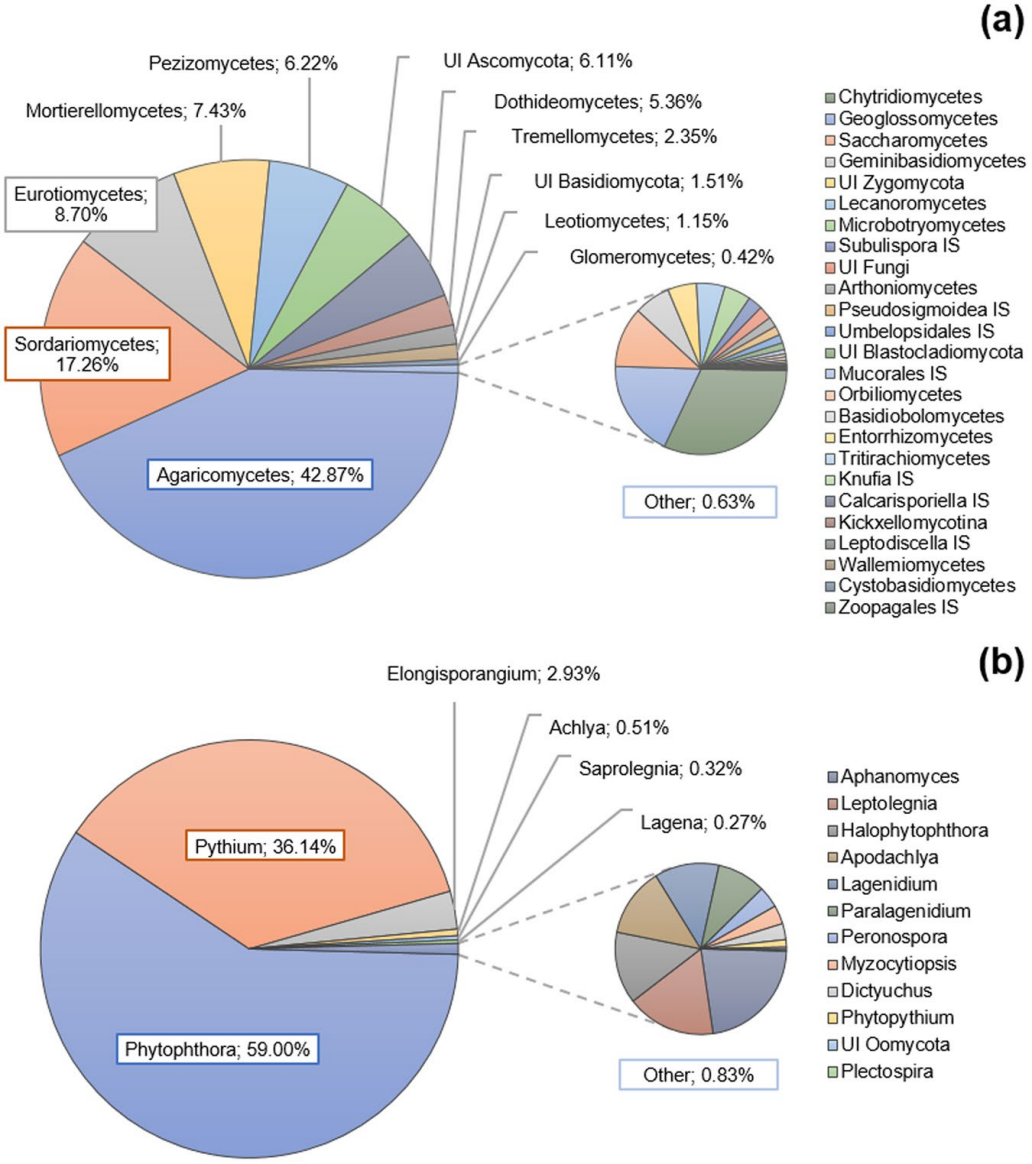
Mean relative OTUs abundance in 26 plots of *Quercus ilex dehesas* of the Andalusian Network for Damage Monitoring in Forest Ecosystems. (**a**) Abundance for fungi OTUs classified by “class” taxonomic level. (**b**) Abundance for oomycete OTUs classified by “genus” taxonomic level. Legends are referred to detail graph (left, “Other” pie chart) ordered by frequency. UI: Unidentified (for those OTUs which classification at the chosen taxonomic level was impossible). IS: Incertidae Sedis. The number of OTUs corresponding to each class (**a**) or genus (**b**) is showed in Supplementary Information, Table S1.

**Figure 2:**
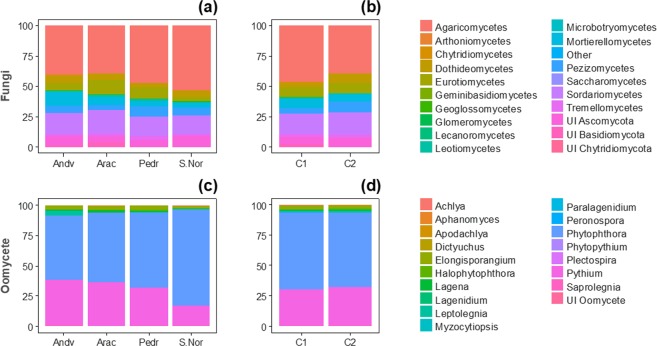
Relative abundance of oomycete and fungal taxa across study zones and mean defoliation class on the 26 studied *Quercus ilex dehesas* of the Andalusian Network for Damage Monitoring in Forest Ecosystems. Vertical axis represents % of frequencies. Fungal abundance for zone (**a**) (%) and mean defoliation class (**b**) (%) is represented for taxonomic level 3 (class). Oomycete abundance for zone (**c**) (%) and mean defoliation class (**d**) (%) is represented for taxonomic level 6 (genus). Andv: Andévalo. Arac: Aracena. S.Nor: Sierra Morena & Sierra Norte. Pedr: Pedroches. UI: Unidentified taxon.

After bioinformatic treatment of the raw data, 1789 fungal OTUs (Operational Taxonomic Units) and 178 oomycete OTUs were confidently assigned to the genus or species level. Among the fungal taxa, Basidiomycota was the most abundant division (46.8%) followed by Ascomycota (45.1%) and Zygomycota (7.4%). The class Agaricomycetes was the most abundant (42.87%) and, together with Sordariomycetes, Eutriomycetes, Mortierellomyetes, and Pezizomycetes, represented the five dominant fungal classes (Fig. 1a). The class Agaricomycetes had its maximum relative abundance in the S.Nor zone (53.2%) and the lowest in Arac (39.4%). The opposite tendency was shown by Mortierellomycetes, which presented their minimum value in S.Nor (3.4%) and their maximum in Andv (11.2%) with a great value in Arac (6.8%) (Fig. 2a). No significant differences appeared for fungal classes in relative abundances between defoliation classes (Fig. 2b). Other relevant classes of Ascomycota were Dothideomycetes (5.36%) and Leotiomycetes (1.15%).

Regarding oomycetes, species of the genera *Phytophthora* and *Pythium* spp. represented over 95% of the overall clustered sequences, followed by *Elongisporangium* spp. (corresponding to *E. undulatum*), with almost 3% of the total sequence abundance, all of them corresponding to plant pathogen species (Fig. 1b). Moving eastward across the plots, the abundance of *Phytophthora* spp. increased and that of *Pythium* spp. decreased, with a significant inverse correlation between the relative abundances of the two taxa (Spearman ρ = −0.966; p < 0.001). However, none of the analysed oomycete taxa presented significant differences in abundance among the defoliation levels (Fig. 2c,d).

The feature classification analysis of the microbiome selected 20 Oomycete OTUs and 349 Fungal OTUs as core features. Among the oomycetes, the most-abundant OTU, present in all the samples with important differences in abundance, was *Phytophthora plurivora* T. Jung & T.I. Burgess (oomycete OTU#1) - which accounted for 15.2% of the total frequencies, except in the case of Arac, where OTU#6 (*Pythium paroecandrum* Drechsler) was the most abundant, representing over 21% of the total frequencies in this zone (Table 1). The fourth-most-abundant oomycete was OTU#3, identified as *Phytophthora quercina* T. Jung & T.I. Burgess, whose presence was greatest in the Andv zone. *Phytophthora cinnamomi* (OTU#4) was the ninth-most-abundant OTU, accounting for 2.2% of the total sequences and identified in 18 of the 26 selected plots. Its presence was greatest in Pedr, where it was the third-most-abundant species (3.5% of the total frequency), and in Andv: in both cases, it showed lower abundance and frequency than *P. plurivora* and *P. quercina*. However, despite the low recovery rate on high-defoliated plots (C2, 38%), the abundance of *P. cinnamomi* in the high defoliated plots in which appeared was significantly high (31.8%).

**Table 1:** Ten most abundant oomycete and fungi OTUs found in 26 *Quercus ilex dehesas* of the Andalusian Network for Damage Monitoring in Forest Ecosystems, ordered by relative frequency (from core-features of each abundance matrix calculated among OTUs present in at least 50% of samples). Freq.: Number of sequences belonging at each OTU relative to the total number of sequences for the considered factor. % of samples: Percentage of samples in which the OTU was identified.

	OTU ID	Taxon	Functional Guild	Location								Average Defoliation				Total (n = 52)	
				Andevalo (n = 16)		S. Aracena (n = 10)		S. Norte (n = 12)		Pedroches (n = 14)		Low (C1) (n = 28)		High (C2) (n = 24)			
				*Freq. (%)*	*% of samples*	*Freq. (%)*	*% of samples*	*Freq. (%)*	*% of samples*	*Freq. (%)*	*% of samples*	*Freq. (%)*	*% of samples*	*Freq. (%)*	*% of samples*	*Total Freq. (%)*	*% of Total samples*
Oomycetes	OTU_1	*s__Phytophthora plurivora*	Plant Pathogen	11.1	100	17.5	100	19.7	100	7.1	100	20.9	100	7.7	100	15.2	100
	OTU_6	*s__Pythium paroecandrum*	Plant Pathogen	6.0	63	21.4	80	0.5	58	0.4	71	11.0	75	11.4	63	7.8	67
	OTU_10	*s__Phytophthora psychrophila*	Plant Pathogen	1.1	69	5.8	40	11.5	75	1.8	57	7.1	67	2.7	50	6.0	62
	OTU_3	*s__Phytophthora quercina*	Plant Pathogen	5.5	100	2.7	90	6.1	75	4.0	86	6.0	92	2.4	81	4.7	89
	OTU_9	*s__Phytophthora citrophthora*	Plant Pathogen	1.5	69	2.9	50	6.7	75	0.4	100	5.2	89	0.5	50	3.5	75
	OTU_17	*s__Pythium heterothallicum*	Plant Pathogen	5.2	56	4.1	80	1.4	75	1.3	86	3.8	78	10.9	75	3.2	73
	OTU_15	*s__Pythium irregulare*	Plant Pathogen	8.7	81	1.7	50	0.7	83	0.1	64	2.7	69	9.5	81	3.0	71
	OTU_7	*s__Elongis-porangium undulatum*	Plant Pathogen	3.9	100	4.6	70	0.4	83	1.3	100	2.9	92	0.8	94	2.6	90
	OTU_4	*s__Phytophthora cinnamomi*	Plant Pathogen	2.0	56	1.9	30	2.1	50	3.5	64	2.1	58	31.8	38	2.2	52
	OTU_16	*g__Pythium sp*.	Plant Pathogen	1.4	75	3.2	30	1.4	67	0.1	57	2.0	64	0.4	50	1.7	60
	Total oomycete core biome frequency			46.4		65.8		50.5		20.0		63.8		78.2		49.9	
Fungi	OTU_3	*s__Mortierella elongata*	Soil-Plant Saprotroph - Endophyte	7.5	100	4.7	100	2.8	100	1.8	100	4.7	100	3.9	100	4.4	100
	OTU_2	*g__Fusarium sp*.	Plant Pathogen-Soil-Plant Saprotroph	4.2	100	3.7	100	3.7	100	1.9	100	3.3	100	3.7	100	3.4	100
	OTU_8	*s__Russula praetervisa*	Ectomy-corrhizal	2.8	100	0.4	90	2.3	100	1.1	79	1.5	92	1.9	94	1.6	92
	OTU_11	*s__Mortierella elongata*	Soil-Plant Saprotroph - Endophyte	2.8	100	1.1	80	2.0	75	6.0	100	1.4	86	1.0	100	1.3	90
	OTU_24	*g__Solicoccozyma sp*.	Unassigned	1.0	100	0.9	100	0.8	100	1.7	100	1.1	100	1.1	100	1.1	100
	OTU_9	*g__Thelonectria sp*.	Soil-Plant Saprotroph	1.4	100	1.1	100	0.8	100	0.6	100	0.8	100	1.4	100	1.0	100
	OTU_25	*g__Penicillium sp*.	Soil-Plant Saprotroph	0.6	100	1.3	100	0.7	100	0.7	100	1.0	100	0.6	100	0.9	100
	OTU_51	*s__Trichoderma spp*.	Soil-Plant Saprotroph	0.6	100	1.1	100	0.5	92	0.7	100	0.8	100	0.7	94	0.7	98
	OTU_48	*s__Clonostachys rosea*	Soil-Plant Saprotroph	0.6	100	1.3	100	0.2	92	0.3	100	0.6	97	0.8	100	0.7	98
	OTU_15	*s__Trichoderma spp*.	Soil-Plant Saprotroph	1.1	94	0.2	100	1.2	92	0.5	93	0.9	92	0.3	100	0.7	94
	Total fungal core biome frequency			22.6		15.8		15.0		15.3		16.0		15.2		15.8	

Two OTUs were identified as *Pythium spiculum* Paul. (OTU#21 and OTU#235), but they were present in only 21.2% of samples, corresponding to 10 plots, with low abundance (1.4% in total), and neither of them were classified as core features.

Among the 349 fungal OTUs present in almost half of the soil samples, fungal OTUs #3 and #11, corresponding to *Mortierella elongata* Linnem., were the most abundant taxon, accounting over 6.1% of the total number of sequences (Table 1). The second most abundant OTU was OTU#2, *Fusarium* spp., present in all the samples analyzed and representing over 3.4% of the total frequency. The third-most-abundant taxon was *Russula praetervisa* Sarnari (OTU#8), present and abundant in all the samples of Andv and S.Nor, but representing only 0.4% of the total abundance in Arac, and identified only in 75% of the samples of Pedr. Its abundance varied significantly with the mean defoliation (F = 3.38; p < 0.01), being higher in plots with low defoliation average (C1).

Two OTUs belonging to the genus *Trichoderma* were classified among the 10 most-abundant species of the core biome: OTU#51, with its maximum frequency in samples from Arac, and OTU#15, representing over 1% of the total number of sequences in samples from Andv and S.Nor. Both OTUs had lower frequency rates in Pedr.

Spearman’s correlation analysis showed a significant negative correlation between the abundance of *Trichoderma* spp. (fungal OTU#51) and that of 10 different OTUs of the oomycete core biome (Table 2), including most of the *Phytophthora* spp., except for OTUs #3 (*P. quercina*), #48 (*Phytophthora* sp.) and #12 (*P. syringae*). Regarding *Pythium* spp., only OTUs #6 (*P. paroecandrum*) and #2 (*Pythium* sp.) presented significant negative correlation. Despite its importance in our results, it was impossible to identify OUT#51 at species level because the well-conserved ITS1 region of *Trichoderma* spp. genus is unable to differentiate between species.

**Table 2:** Significant correlations between the abundance of OTU#51 (*Trichoderma* spp.) and the abundance of the core biome OTUs of oomycete dataset at 26 *Quercus ilex dehesas* of the Andalusian Network for Damage Monitoring in Forest Ecosystems. Rank: Order of the OTUs in the core features classification by abundance (1 for the most abundant, to 20 for the less abundant). ρ: Spearman’s Rho value. *Significant correlation at p < 0.05; **Significant correlation at p < 0.01.

Rank	OTUs	Assigned Taxon	Ρ
1	OTU#1	*P. plurivora*	−0,392**
2	OTU#6	*P. paroecandrum*	−0,263*
3	OTU#10	*P. psychrophila*	−0,272*
5	OTU#9	*P. citrophthora*	−0,378**
8	OTU#7	*E. undulatum*	−0,325*
9	OTU#4	*P. cinnamomi*	−0,345**
12	OTU#5	*P. cactorum*	−0,277*
13	OTU#8	*P. gonapodyides*	−0,308*
14	OTU#2	*Pythium* sp.	−0,395**
15	OTU#14	*P. cambivora*	−0,379**

### Functional guilds

Of the OTUs confidently classified in an ecological guild (Table 3), the *Soil-Plant Saprotroph* guild was the most abundant in all cases (over 15%), except in S.Nor, where *Plant Pathogen* was the most-abundant guild (over 21%). However, when the frequencies of all the putative plant pathogenic OTUs were summed (including *Plant Pathogen*, *Opportunistic Pathogenic Species* and those OTUs classified in >*1 Guild*, which includes *Plant Pathogen* as one of those guilds), their abundance was greater than that of the rest of the guilds, in all the zones studied.

**Table 3:** Mean ± SE relative abundance of functional guilds between study zones and defoliation levels of *Quercus ilex dehesas* of the Andalusian Network for Damage Monitoring in Forest Ecosystems. The data of functional guilds which presented significant differences (ANOVA with Bonferroni mean differences test, or Kruskal-Wallis rank sum test) were written in Italic. Andv: Andévalo. Arac: Aracena. S.Nor: Sierra Morena & Sierra Norte. Pedr: Pedroches. C1: Low average defoliation plots. C2: High average defoliation plots. t_(df)_^sign^ = t-value from the Student test with the degree of freedom and the significance level of the test. ^†^Only one sample of Andevalo presented confident identification of endophyte organismSignificance level: *p ≤ 0.05; **p ≤ 0.01; ***p ≤ 0.001; n/sp > 0.05. Values with the same letter are not significantly different (p > 0.05). ^(††)^Levene test p < 0.05 (variances were different between factors).

Functional Guild	Location				Total	Defoliation		t_(df)_^sign.^
	Andv	Arac	S.Nor	Pedr		C1	C2	
>1 Guild designation	3.3 ± 0.1	3.6 ± 0.2	3.8 ± 0.2	4.0 ± 0.3	3.6 ± 0.1 ^n/s^	3.7 ± 0.1	3.6 ± 0.2	0.46 _(50)_^n/s^
Animal Pathogen	*0.9* ± *0.1*^*b*^	*1.0* ± *0.1*^*ab*^	*0.8* ± *0.1*^*b*^	*1.3* ± *0.1*^*a*^	***1.0*** ** ± ** 0.1**	*0.9* ± *0.1*	*1.2* ± *0.1*	***−2.10***_***(52)***_ *
Arbuscular Mycorrhizal	3.4 ± 0.5	3.3 ± 0.4	5.1 ± 0.9	3.5 ± 0.9	3.8 ± 0.3 ^n/s^	3.9 ± 0.4	3.5 ± 0.5	0.44 _(50)_^n/s^
Dung Saprotroph	*1.0* ± *0.1*^*ab*^	*0.7* ± *0.1*^*c*^	*0.8* ± *0.1*^*bc*^	*1.1* ± *0.1*^*a*^	***0.9*** ** ± ** 0.0***	0.9 ± 0.1	0.8 ± 0.1	0.61 _(50)_^n/s^
Ectomycorrhizal	*9.9* ± *0.7*^*a*^	*9.8* ± *0.9*^*a*^	*9.4* ± *0.4*^*a*^	*5.5* ± *0.6*^*b*^	***8.7*** ** ± ** 0.4***	9.0 ± 0.5	8.0 ± 0.8	1.08 _(51)_^n/s^
Endophyte	0.2^†^	*0.14* ± *0.02*^*b*^	*0.12* ± *0.02*^*b*^	*0.4* ± *0.1*^*a*^	***0.22*** ** ± ** 0.04**	0.22 ± 0.04	—	—
Fungal Parasite	0.24 ± 0.02	0.31 ± 0.04	0.4 ± 0.1	0.29 ± 0.04	0.30 ± 0.02 ^n/s^	0.29 ± 0.03	0.33 ± 0.03	−0.81 _(49)_^n/s^
Lichenized	0.3 ± 0.0	0.25 ± 0.04	0.24 ± 0.03	0.19 ± 0.04	0.25 ± 0.02 ^n/s^	0.24 ± 0.02	0.28 ± 0.06	^(††)^−0.64 _(12.6)_^n/s^
Oportunistic species	3.7 ± 0.2	3.4 ± 0.2	3.9 ± 0.2	4.1 ± 0.3	3.8 ± 0.1 ^n/s^	3.8 ± 0.1	3.7 ± 0.3	0.52 _(54)_^n/s^
Plant Pathogen	10.0 ± 1.0	14.1 ± 5.9	21.7 ± 8.4	13.4 ± 6.1	14.5 ± 2.8 ^n/s^	15.6 ± 3.4	11.9 ± 5.0	0.59 _(50)_^n/s^
Soil-Plant Saprotroph	*15.7* ± *0.5*^*ab*^	*15.4* ± *0.5*^*ab*^	*14.0* ± *0.5*^*b*^	*17.1* ± *0.7*^*a*^	***15.6*** ** ± ** 0.3**	15.3 ± 0.4	16.4 ± 0.5	−1.71 _(50)_^n/s^
Unclassified	51.7 ± 0.7	50.5 ± 3.4	45.2 ± 4.9	51.4 ± 4.0	49.8 ± 1.7 ^n/s^	49.0 ± 2.0	52.0 ± 3.2	−0.79 _(54)_^n/s^

Five functional guilds showed significant differences among zones, the *Ectomycorrhizal* (EcM) and *Endophyte* guilds exhibiting opposing trends, with the minimum frequency values of EcM species corresponding to the zone with maximum values for *Endophyte*. In the Pedroches zone (Pedr) the maximum values were found for the *Endophyte*, *Animal Pathogen*, *Dung Saprotroph*, and *Soil-Plant Saprotroph* functional guilds, with a significantly-lower value for EcM. Only the *Animal pathogen* guild showed significant differences among defoliation levels, and no *Endophyte* species was identified for high defoliated ones.

**Figure 3:**
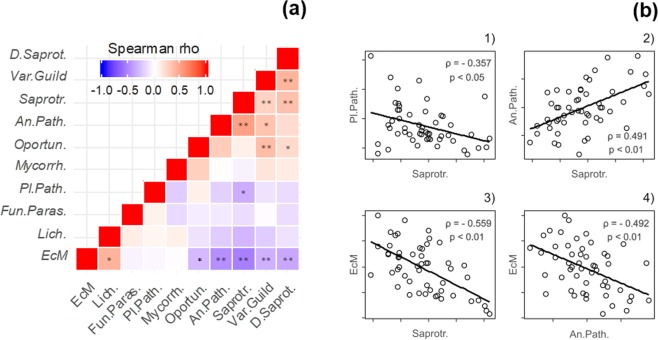
Relationships between functional guilds on the 26 studied *Quercus ilex dehesas* of the Andalusian Network for Damage Monitoring in Forest Ecosystems. (**a**) Correlation matrix between functional guilds relative abundance in each sample. *Significant correlation at p < 0.05; **significant correlation at p < 0.01. (**b**) Scatterplots of significant correlations between Functional Guilds: (1) Plant Pathogen/Soil-Plant Saprotroph; (2) Animal Pathogen/Soil-Plant Saprotroph; (3) Ectomycorrhizal/Soil-Plant Saprotroph; (4) Ectomycorrhizal/Animal Pathogen. ρ: Spearman’s Rho value. p: Significance level of correlation.

The Spearman’s correlation matrix between functional guilds (Fig. 3a) shows a significant, positive correlation of *Soil-Plant Saprotrophs* with *Animal Pathogen* abundance (ρ = 0.491, p < 0.01) and a negative one with *Plant Pathogens* (ρ = −0.356, p < 0.05) (Fig. 3b-1 and 2).

**Figure 4:**
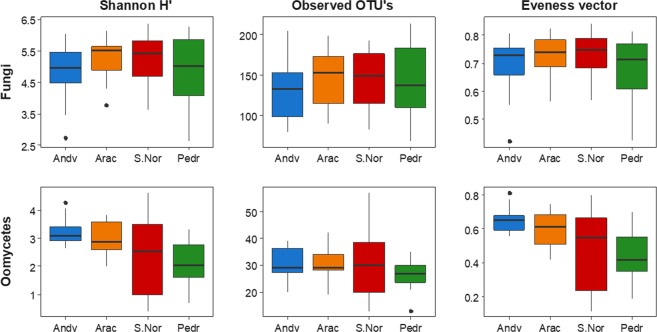
Analysis of Shannon H’ diversity, (left), OTUs richness (centre) and Pielou-e eveness vector (right) by geographical zones, for the studied *Quercus ilex dehesas* of the Andalusian Network for Damage Monitoring in Forest Ecosystems, for fungi (up) and oomycete (down).

On the other hand, EcM was negatively correlated with *Soil-Plant Saprotrophs* (ρ = −0.559, p < 0.01; Fig. 3b-3), *Animal Pathogen* (ρ = −0.492, p < 0.01) (Fig. 3b-4), *Dung Saprotrophs* (ρ = −0.384, p < 0.01), *Opportunistic Pathogenic Species* (ρ = −0.348, p < 0.05) and >*1 Guild*, (ρ = −0.343, p < 0.01). The *Dung Saprotroph* guild was positively correlated with *Soil-Plant Saprotrophs* (ρ = 0.332, p < 0.01) and with the species assigned to more than one guild (>*1 Guild*, ρ = 0.392, p > 0.01). Other significant correlations appeared between >*1 Guild* OTUs, *Lichenized* and *Opportunistic Pathogenic Species*.

### Fungal and oomycete diversity

The Good’s Coverage indices for the selected thresholds were high for the rarefied matrices of the oomycetes (G = 0.98) and fungi (G = 0.99), indicating that over 98% of the identified OTUs were sampled in the rarefaction process and thus included in the α-diversity analysis.

The Shannon H’ and Pielou Evenness α-diversity indices of the oomycetes exhibited similar trends according to the geographical longitude, their abundances decreasing from west to east (Fig. 4), with significant differences between Pedr and Andv (H_Shannon_: 5.69, p < 0.01; H_Evenness_: 6.06; p < 0.01), but without differences in OTUs richness. The fungal community did not show significant differences for the α-diversity indices. When these indices were compared between the mean defoliation levels for both communities, only oomycete evenness was significantly lower for the greatly-defoliated plots (χ^2^: 4.64, DF: 1, p < 0.01); the fungal community did not show differences.

**Figure 5:**
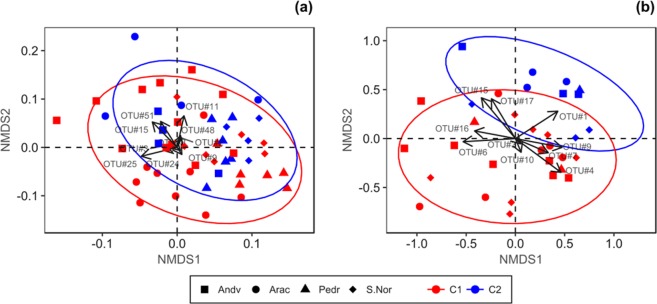
Non-metric multidimensional scaling analysis of OTUs abundance influence in defoliation levels and site location, for the 26 studied plots on *Quercus ilex dehesas* of the Andalusian Network for Damage Monitoring in Forest Ecosystems. (**a**) Analysis for fungi dataset. (**b**) Analysis for oomycete dataset. Ellipsoid indicates normal probability of point distribution at 90% confidence level for mean defoliation classes of plots. Arrows show influence vectors for the ten most abundant OTU’s of fungi and oomycete dataset.

Regarding β-diversity, ANOSIM showed the influence of location in the composition of the fungal community (R = 0.167; p < 0.001), whereas for the oomycete community (R = 0.078; p < 0.05) differences were only found between Pedr and Andv (R = 0.214; p < 0.01) and between Pedr and S.Nor (R = 0.125; p < 0.05). The non-linear metrics analysis (NMDS) showed a more-aggregated zonal cluster for the fungal community, in comparison with the oomycetes (Fig. 5), but also a clearer separation of the high-defoliated plots for the oomycete community. Oomycete OTU#1 (*P. plurivora*) had the vector which most clearly influenced the separation between defoliation classes.

## Discussion

To the best of our knowledge, this work is the first study of soil biodiversity in holm oak *dehesas* ecosystems, exploring the interaction between fungal and oomycete communities and their influence in the decline symptoms, focusing on the soil microbiome. *Phytophthora* spp. dominated the oomycete community, but the species usually related to *Q. ilex* root rot (*P. cinnamomi* and *P. spiculum*) did not appear among the most abundant, nor were they related directly to defoliation levels.

It must be considered as a limitation of our study the lack of specific data from the rhizosphere. Without this information, the discussion on the shifts of an important guild like Endophytes could not be addressed, but despite holm oak rhizosphere was not analysed, the revision of the ecological roles of the key taxa identified in this work lead us to believe that most of them should be involved in the plant-microbiota interactions, agreeing with the results of other authors^48,49^. Our results showed that the fungal community of declined *dehesas* includes a wide range of plant pathogens and key taxa of EcM and *Trichoderma* spp. which would be related to the tree health status. The metabarcoding analysis revealed that structural changes of the fungal community and the diversity level of the oomycete community were related with the mean defoliation of the holm oak *dehesas* analyzed, agreeing with our work hypothesis.

### The fungal community of *dehesas* has a wide range of plant pathogens and abundance of EcM

Despite the large number of fungal phytopathogens described in the studied soils, it is not possible to determine the direct influence of these species on the health status of the trees. For example, some of the fungal pathogens identified in our samples have been found previously in *dehesas* as a part of the endophytic mycobiota of pasture species, such as species from the genus *Colletotricum* spp., *Fusarium* spp., *Sordaria* spp. or *Alternaria* spp.^50^. However, the Dothideomycetes *Alternaria alternata* and *Pyrenochaeta cava* were isolated from *Quercus robur* roots by Kwaśna *et al*.^51^, presenting significant changes in abundance according to the degree of tree defoliation. Therefore, the increment in the abundance of some fungal pathogens, such as Dothideomycetes, in the high-defoliated plots, might be due to the pathogenicity of some species, but also to changes in the functionality of mycobiota related to the understory or grassland species.

*Mortierella elongata* is a saprotroph often isolated in natural soils, being related their abundance with soil improvement in nutrient quality and fertility^52,53^. It plays an important role in the degradation of several compounds, including toxic ones^54^, and its abundance can be considered an indicator of the resilience level of *dehesas* ecosystems. Of the symbiotic species, *Russula* was the most-abundant genus, with 76 different OTUs classified within it, including the third-most-abundant species, *R. praetervisa*. Ectomycorrhizal abundance and biodiversity have been related to fine root vitality in *Quercus robur* forests, being considered the most-important below-ground trait related to their decline^55^. Also, its abundance has been related to benefits for the tree health status and the establishment of *Quercus* spp. seedlings^56^. In our work, we did not find a relationship between Russulaceae abundance and the individual pathogenic *Phytophthora* spp., but statistically-significant differences were found when *R. praetervisa* abundance was analysed in relation to defoliation, both results agreeing with other studies in *Q. ilex* forests^22,57^.

In our search for plant pathogens within the core biome of the fungal community, the genus *Fusarium* was the most relevant. This genus includes several soil-borne plant pathogens, related to forest and agricultural plant diseases in woody, shrub and herbaceous hosts^58^. The *F. oxysporum* species complex causes vascular wilts, damping-off and crown and root rot in a wide range of species, being able to produce the death of holm oak seedlings in a pathogenicity test^59^. The role of *F. eumartii* in the decline of *Q. robur*, *Q. cerris* and *Q. pubescens* in Italy has been recognised^60,61^, and other species of this genus have been linked with diseases in other *Quercus* spp.^62–64^. In addition to *Fusarium* spp., 39 more OTUs within the fungal community core biome were assigned to plant pathogenic species, including some species known to be pathogens of oaks - such as some *Peniophora* spp.^65^ (among which, OTU#816 was identified in 98% of samples), *Pyrenochaeta* spp., *Thelonectria* spp., *Alternaria alternata*^66^, *Sporothrix inflata, Pezicula radicicola*^51^ and *Phomopsis* spp.^67^. The abundance of fungal pathogenic species in zones with a low H’ index for oomycetes, such as Pedr and S.Nor, coincided with a lower presence of beneficial fungal species, such as strict saprotrophs, AM or EcM. These circumstances could lead to an environment that is ideal for triggering the decline of trees.

### *Phytophthora* spp. dominate the oomycete community in declining *dehesas*

*Phythophthora* was the dominant genus among oomycetes, being the most-abundant species *P. plurivora*, instead of those related to holm oak root rot according to the literature – *P. cinnamomi* and *P. spiculum*^16,17,68^. Particularly, *Pythium spiculum*, considered a trigger of holm oak decline and mortality^69^, was excluded from the oomycete core biome. Among the oomycete pathogens causing root rot in *Quercus ilex, P. psychrophila*, by its abundance, and *P. quercina*, by its frequency of appearance, were the most-important taxa, *P. cinnamomi* appearing as the ninth-most-abundant oomycete, present in 52.7% of samples. However, *P. cinnamomi* presented a very high frequency in the samples of high-defoliation plots (C2) in which it was identified, agreeing this result with the great susceptibility that holm oak presents against this pathogen^70^.

*Phytophthora plurivora* is a very-common soil-borne pathogen that has been isolated from soils of many natural forests in Europe. It was recorded as the most-abundant species in a survey of oak stands in Italy^9^ and in chestnut forests of central Italy, in a study of soil *Phytophthora* biodiversity through metabarcoding^40^. Its pathogenicity on *Quercus alba*^71^, *Q. robur*, *Q. petraea, Q. rubra*^72^ and *Q. cerris*^73^ is recognised. However, to our knowledge, there is no evidence of a direct pathogenic relationship with *Q. ilex* root rot, or of its involvement in the decline syndrome in *dehesas*, the work of Vettraino *et al*.^9^ being the only one that recorded this species (designated as *P. citricola*) under *Q. ilex* trees (subsp. *ilex*). The abundance of this species in our samples was significantly high in low-defoliated stands, which influenced NMDS aggregation of defoliation classes. The high recovery rate of *P. plurivora* and its relationship with the defoliation encourage the study of their involvement in holm oak decline.

Most of the *Phytophthora* taxa found in our samples were also recorded in a soil analysis in the search for oomycete in natural *Q. ilex* forests of eastern Spain^29^, where *P. psychrophila* and *P. quercina* were the most-relevant taxa, and also a qPCR survey showed *P. quercina* abundance in *dehesas* of Extremadura^30^; *P. psychrophila* and *P. quercina* appeared, respectively, as the third- and fourth-most-abundant oomycete species in our study. Both taxa have been confirmed as *Q. ilex* root pathogens, causing root rot and tree death^74^. However, in the work of Català *et al*.^29^, no match was found for *P. plurivora* and *P. cinnamomi*. The overall specific composition of the *Phythophtora* spp. community in our samples was more similar to that described by Vettraino *et al*.^9^, who found *P. plurivora*, *P. quercina*, *P. cinnamomi* and *P. cactorum* – in that order – as the most-frequent *Phytophthora* spp. isolated in stands of *Q. ilex* affected by oak decline.

Regarding the severity of symptoms associated with the oomycete community, no significant differences in taxa abundance were found among the defoliation categories, agreeing with the works of Corcobado *et al*.^22,57^. Notwithstanding, defoliation and the severity of root rot should be related^68^; in our case, this relationship was detected for the overall structure and functionality of the oomycete community (diversity and evenness), including the influence of the fungal community (EcM abundance).

Given our results, the involvement in the oak decline of all the plant pathogens present in the *dehesa* soils should be considered, not only that of *P. spiculum* and *P. cinnamomi*, but also other pathogenic *Phytophthora* species - such as *P. plurivora*, *P. quercin*a and *P. psychrophila* - and other fungal plant pathogens such as *Fusarium* spp. or other phytopathogenic taxa belonging to the class Dothideomycetes.

### EcM and AM are key guilds related to shifting soil microbiota functionality in *dehesas* ecosystems

The *Ectomycorrhizal* (EcM) and the *Arbuscular Mycorrhizal* (AM) guilds, the third- and fourth-most-abundant functional guilds, respectively, had lower abundance rates overall when compared with other works in soils of different natural ecosystems^45,46^. *Quercus ilex* is a species that often presents mycorrhizal association with different species of Ascomycetes and Basidiomycetes^75^. A significant reduction in EcM and increment in plant pathogens (including *Plant Pathogen* and *Opportunistic Species* guilds) were found in Pedr, coinciding with its status as the most-high-defoliated zone, being the higher EcM abundance correlated with lower pathogen and saprobe abundance, agreeing our results with other works^76^. Corcobado *et al*.^57^ identified non-mycorrhizal root tips of *Q. ilex* as vulnerable points for pathogen invasion. Beneficial mycorrhizae, including AM and EcM, can potentially induce higher pathogen tolerance^77^ through stimulation of the plant immune system^33^. Therefore, either the increase of the root uptake and the effect on plant immune response might be related with lower defoliation level of trees in zones with high EcM and AM abundance.

The *Animal Pathogen*, *Soil-Plant Saprotrophs*, and *Dung Saprotroph* guilds had their maximum values in Pedr, which showed the lowest α-diversity values for oomycetes and an increment in the abundance of *Phytophthora* spp., with good values for the diversity of the fungal community, but lower EcM abundance. This is another example of the greater influence of the community structure and functionality, in comparison with the specific composition. It also supports the existence of a triple relationship among the abundance of EcM and other beneficial fungi, the plant pathogens and the tree health status, agreeing with other works^22,57^.

### High diversity and changes in community structure are associated with the severity of symptoms

In comparison with recent analyses of the mycobiota in soils of different ecosystems^45,46,78^, the *dehesas* had a high Shannon H’ index (greater than 4.5 in all the studied zones) and a high value of evenness (exceeding 0.7). This shows a high diversity and good equilibrium of the mycobiota, but with a high proportion of plant pathogens and significant changes in the specific composition of the soil community among zones, agreeing with the results of ANOSIM. No relationship of the studied parameters with plant community changes was found, agreeing with other authors who reported that changes in the diversity of mycobiota were decoupled from plant α-diversity^45,46^.

The values of the α-diversity indices of the oomycete community were, in general, higher than those found by Català *et al*.^29^ in natural forest soils of eastern Spain and were similar to (slightly higher than) those reported by Vannini *et al*.^40^ for chestnut forests of Italy. The lowest values of the Shannon index and evenness vector for the oomycete community occurred in Pedr, the most-defoliated zone, showing low species richness and differences in taxa abundance, agreeing with the significantly-lower values of evenness in the highly-defoliated plots. Although a direct relationship between specific oomycete taxa, or their abundance, and the severity of symptoms was not found, the β-diversity analysis showed aggregation of high-defoliated trees regarding the oomycete community. Similar results were obtained by Linaldeddu *et al*.^79^ when evaluating the diversity of the fungal endophytic community in declining *Quercus suber* L. trees. The severity of defoliation, therefore, seems to be related more to the functional structure of the oomycete community – driven by the evenness - than to its specific composition, being presented the change in the specific composition of the fungal community and the reduction in competition between root pathogens as the most influential drivers related with decline symptoms.

### The abundance of *Pythium* spp. and *Trichoderma spp*. influencing the low *Phytophthora* spp. dominance

The abundances of *Pythium* and *Phytophthora* influenced each other, but it remains unclear how the changes were influenced by location or by a natural equilibrium between the taxa. Our results show a significant decrease in oomycete α-diversity in soils of highly-defoliated stands, coinciding with an increase in the abundance of *Phytophthora* spp. Other authors have demonstrated that the mixture of *P. cinnamomi* with different species of *Pythium* or other pathogenic fungi (such as *Fusarium oxysporum*) did not increase the severity of symptoms^59^, supporting the hypothesis that, in soils with less diversity of oomycetes, *P. cinnamomi* and other pathogenic *Phytophthora* species have an increased ability to infect roots and affect the health status of the tree, increasing the severity of visible symptoms.

Another key relationship between taxa was the significant inverse correlation between OTU#51 abundance (*Trichoderma* spp.) and most of the core biome taxa of oomycetes in the studied plots (Table 2). The effects of *Trichoderma* spp. in biocontrol strategies are well known^80^. *Trichoderma asperellum*, *T. hamatum*, and *T. virens* have been isolated from holm oak roots (*Q. ilex* subsp. *ilex*) and reduced the root rot of seedlings in a controlled experiment including *P. nicotianae* and *P. cinnamomi*^34^. Other *Trichoderma* spp. have also been proved to be antagonists of diverse *Phytophthora* spp.^81–85^. The evidence of the multiple effects of *Trichoderma* spp. against multiple phytopathogenic species should encourage further investigation of this relationship in the holm oak *dehesas*. The next steps in the search for control strategies based on the management of fungal soil biota should be the isolation of *Trichoderma* spp. from the studied soils to identify the fungus at species level, together with the analysis of root endosphere, which would provide useful information about the endophyte behaviour of the present species, as it has been shown for other *Trichoderma* spp.^86,87^.

### Metabarcoding is a powerful tool in the management of soilborne diseases

Biocontrol strategies are considered environmentally friendly approaches in the management of plant diseases, but the high complexity of ecological interactions between the microbiome components could lead to negative impacts on non-target organisms^88^, and the misunderstand the mechanism of the interactions provokes the inefficiency, or even adverse effects of treatments^89^. Thus, the study of the microbiome is an important step to develop management strategies to reduce tree decline damage through biocontrol strategies, and the knowledge about microbial community has demonstrated to have important implications in those aspects^47^. Metabarcoding techniques could help to focus biocontrol strategies, searching for the adaptability and synergism of new products with the indigenous microbiome, or characterizing microbiota of disease-suppressive soils and the role of key taxa in this microbiome. In this sense, further studies confirming the presence of the identified key taxa in the rhizosphere are encouraged. Additionally, it is recommended to complement metabarcoding approaches with other methods, beyond the rhizosphere microbiome analysis, to better understand the role of the most relevant taxa, including those taxa that are identified for the first time associated with this system. The identification of relationships between *Trichoderma* spp., EcM and *Phytophthora* spp. in soils related to holm oak decline leads to new soil management strategies in those stands affected by root rot, directly linked to the microbiome ecology. Moreover, biological control strategies must consider the influence of location and environmental variables in soil community^90^, being the metabarcoding analysis an adequate technique to provide this information.

## Conclusion

This work is a novel contribution to the study of soil biodiversity in holm oak *dehesas* ecosystems, analysing for the first time the fungal and oomycete communities together in *dehesa* soils. Our results revealed high levels of diversity in the fungal community and medium to low diversity of oomycetes, the presence of key taxa influencing holm oak health status and oomycete pathogenic abundance, and the dominance of plant pathogens and saprotrophs in the analysed soils. The microbiome presented significant influence in the defoliation status, confirming our main hypothesis, and suggesting that both the structure of the fungal community and the level of diversity of the oomycete community were related with the tree health status.

On the other hand, the high degree of soil microbiome diversity in declined *dehesas* revealed on this work encourages to a deeper research into the relationship between different taxa, considering a tripartite relationship (plant-pathogens-other taxa)^47^, but also in the study of the influence of environmental factors in the soil community, beyond the simple picture of a single plant-pathogen interaction. The findings about the presence of key taxa related with defoliation symptoms or pathogen abundance, must drive us to consider biocontrol strategies in the management of the holm oak root rot, through methods and applications that respect the local microbiota. Several biocontrol strategies could be explored deriving from our results, such as the application of *Trichoderma* spp. to control the inoculum of pathogenic oomycetes, as well as the application of mycorrhizae as a strategy to improve the health status of trees, but further research is needed to clarify how both key taxa interacts between them and with holm oak.

## Material and Methods

### Study area

**Figure 6:**
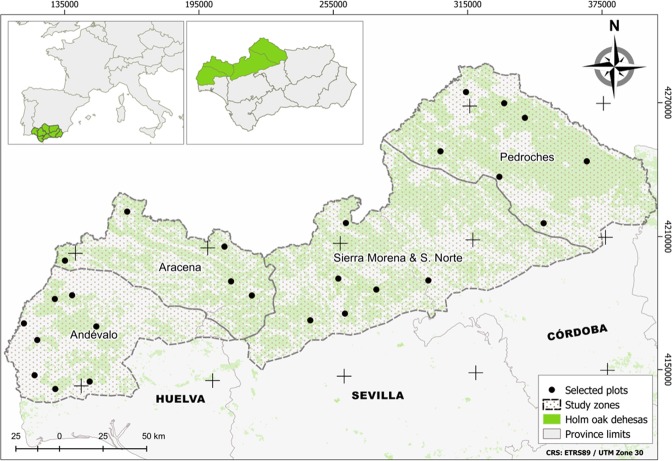
Map of sampled plots of *Quercus ilex dehesas* of the Andalusian Network for Damage Monitoring in Forest Ecosystems.

This study was focused on holm oak (*Quercus ilex* subsp. *ballota*) *dehesas* of north-west Andalusia (Fig. 6). The studied area was divided into four zones - Andévalo (Andv), Sierra de Aracena (Arac), Sierra Norte de Sevilla and S. Morena (S.Nor) and Valle de los Pedroches (Pedr) - located in the provinces of Huelva, Sevilla and Córdoba, representing the core area of the holm oak *dehesa* ecosystems of Andalusia (Fig. 6). This division was made based on differences in environmental factors and silvicultural characteristics (Table 4).

**Table 4:** Mean values of environmental and silvicultural parameters considered for the selection of the study zones (m.a.s.l.: meters above sea level). Average values for the period 1969–2000, taken from the Andalusia Environmental Information Network - REDIAM; Consejería de Medio Ambiente, Junta de Andalucía (http://www.juntadeandalucia.es/medioambiente/site/rediam/portada/). The average tree cover, the dominant tree age and the prevalent class of dehesa were determined as the trend of all the visited plots for each zone, following the criteria of Costa Pérez *et al*.^104^. *Low = N < 50 tree ha^−1^; Medium = 50 < N < 80 tree ha^−1^; High = N > 80 tree ha^−1^. **Young trees = 50–90 years/20–40 cm Ø; Mature trees = 110–210 years/50–80 cm Ø; Old trees = 210–250 years/90–110 cm Ø; Very old = > 250 years/Ø > 110 cm.

	Andevalo	Aracena	Sierra Morena & S. Norte	Pedroches
*Mean Precipitation (mm)*	675.7	829.2	712.1	572
*Mean Annual temperature (°C)*	17.4	16.2	16.1	15.8
*Mean Evapotranspiration (mm)*	1445	1396	1458	1443
*Max elevation (masl)*	293	1046	936	943
*Mean elevation (masl)*	191	434	429	570
Average cover*	Low	Medium	High	Low
Dominant tree age**	Young to Mature	Mature	Young	Old to very old
*Class of dehesa*	Cultivated dehesa	Normal tree cover with shrubs and pasture	High tree cover with shrubs and pasture	Pastures

A total of 26 plots were selected among the permanent environmental monitoring points of the Andalusian Network for Damage Monitoring in Forest Ecosystems (Red de Información Ambiental, Consejería de Medio Ambiente y Ordenación del Territorio - Junta de Andalucía, ICP Forest, EC Network level II - www.juntadeandalucia.es). The plots were selected based on the following criteria; (i) >85% of the trees were *Q. ilex*, (ii) a positive record of *P. cinnamomi* and (iii) the tree mortality rate. To calculate the mean plot defoliation, all the trees of the plots (24 trees × 26 plots) were assessed for defoliation following the methodology of Eichhorn *et al*.^91^. The mean defoliation for each plot (%), calculated using the individual defoliation of the 24 trees per plot, ranged between 13.3% and 56.9% resulting in 14 plots classified as C1 (mean defoliation < 25%), and 12 plots classified as C2 (25% ≤ mean defoliation < 65%) (Supplementary Information, Table S2). The mean defoliation showed significant differences among the study zones (χ^2^ = 11.1; DF = 3; p < 0.05), the highest value corresponding to Pedr.

### Soil sampling

In each plot, soil samples were collected from the rhizosphere under the crown of two trees with defoliation degree similar to the mean defoliation of plot (28 samples under C1 trees and 24 samples under C2 trees, n = 52), searching for the representability of the mean plot conditions (soil, exposure and slope). For each tree selected, sub-samples were taken under the crown for two orientations (North and South) and two distances from the trunk (0.5 m and 1–2 m), making four sub-samples per tree. Then, the sub-samples of each tree were bulked and stored at 4 °C until processing. Samples were collected from November to December 2015. The soil samples were processed within the next 24 hours following their collection; soil was dried at room temperature for 24 h and well-mixed, litter and roots were separated, and 20 g of the fine fraction was collected after passing the soil through a 1-mm sieve.

### DNA extraction, ITS1 amplification, library generation, and sequencing

Total DNA was extracted from soil using the MoBIO Power Soil DNA Isolation kit (MoBIO Laboratories, Inc., CA, USA). Five replicates of fresh soil (0.33 g each) were taken from each soil sample. The aliquots were mixed together, and total DNA was purified and concentrated using a DNA purification kit (Wizard SV Genomic DNA Purification System; Promega Corporation, WI, USA). The DNA concentration was quantified, with a Qubit dsDNA BR Assay kit (Thermo Fisher Scientific Inc., MA, USA), prior to amplification.

Additionally, two mock communities were created by pooling DNA from a pure culture of 13 different species of oomycetes and another with seven species of fungi (Supplementary Information, Table S3); one community of each taxa had the same concentration of each species (Oo= and F=) and the other had different concentrations (Oo≠ and F≠). These were used as controls and were processed in the same way as the samples, following the methodology of C. Morales Rodriguez (Unpublished).

The internal transcribed spacer 1 (ITS1) was amplified in a multiplexing PCR using a set of barcoded primers: ITS1F and ITS2^92^ for fungi, and ITS6 and ITS7 for oomycetes^93^. The primers were tagged with different barcodes to distinguish different samples. Moreover, 3 replicates of each control community were amplified with different barcoded primers of the ITS1F/ITS2 and ITS6/ITS7 primer pairs (Supplementary Information, Tables S4, S5). Negative controls and cross-reactions (Oo with ITS1F/2 and F with ITS 6/7) did not originate any PCR product.

The PCR reaction mixture consisted of 25 μl of Maxima Hot Start PCR Master Mix (Thermo Fisher Scientific, USA), 2 μM of each primer, 4 µl of 2 mg ml^−1^ BSA and 3 μl of DNA template diluted to 50 ng µl^−1^, in a total volume of 50 μl. The thermal cycle was an initial 5-min step at 95 °C, followed by cycles (30 for fungal ITS and 35 for oomycete ITS) of 40 s of denaturation at 95 °C, 2 min of annealing (58 °C for fungal ITS amplification and 60 °C for oomycete ITS) and 1 min of extension at 72 °C, with a final extension step of 7 min at 72 °C. For each sample and the controls, three replicates of PCR for fungal ITS and five for oomycetes ITS were carried out and the products were pooled.

Amplicons were purified using the MagJet NGS Cleanup and Size Selection kit (Thermo Fisher Scientific Inc., MA, USA) and the final concentration of purified PCR product was quantified using a Qubit dsDNA HS Assay kit (Thermo Fisher Scientific Inc., MA, USA). The resulting amplicons were pooled in equal amounts to give a total volume of 40 µL, with a concentration of 200 ng µL^−1^, and were sent to Eurofins Genomics AT GmbH (Vienna, Austria), to be sequenced in an Illumina MiSeq 2 × 300 bp platform. Both fungal and oomycete libraries were included on the same Illumina run. The reads are available in the EMBL Nucleotide Sequence Database (http://www.ebi.ac.uk/embl) under the project name “NGS_Dehesa” and accession number PRJEB28448.

### Sequence processing, OTU selection, and abundance matrix construction

A bioinformatic pipeline was adapted from Bálint *et al*.^94^ and Sapkota & Nicolaisen^95^. The primers were debarcoded and classified by Eurofins Genomic AT GmbH (Vienna, Austria). The sequences were joined (22 774 511 sequences) and reads with mismatches or ambiguous bases were unassigned (1 246 294 sequences). Finally, only paired sequences in which the 5′ barcode and forward primer and 3′ barcode and reverse primer were found, were used in the bioinformatic analysis (Supplementary Information, Table S6). Singletons were excluded from the analysis.

The forward and reverse sequences of paired reads were trimmed, the primers were eliminated, and sequences were overlapped and paired using CLC Genomics Workbench 3.6.5 (https://www.qiagenbioinformatics.com/) (Supplementary Information, Table S7). The ITS1 region was extracted with the Fungal ITS Extractor version 2^96^, sequences were clustered in operational taxonomic units (OTUs) and the chimeric sequences were filtered - de-novo chimera filtering for oomycete files - using USEARCH 8.1^97^ with a similarity level established at 98%. The consensus sequences of the OTUs were identified with the GenBank nucleotide database (nr/nt) of NCBI, using the Megablast algorithm, and the results were parsed, and the sequences retained in MEGAN 6.10.8^98^ to assign taxonomic identifications to each OTU (Supplementary Information, Table S8). In some cases, ITS1 was unable to differentiate between species belonging to genera which presented a high conserved ITS1 region, such as *Trichoderma* spp., but after a thorough supervision of the taxonomy, the metabarcoding analysis showed enough accuracy to study the diversity and composition of the fungal and oomycete communities, clearly differentiating between genera in all cases. Afterward, abundance tables of OTUs for oomycete and fungal files were constructed using USEARCH 8.1 with a level of identity of 98%.

The amplified ITS sequences from all the species included in the internal controls were correctly identified. The mean frequency of debarcoded paired sequences did not differ between oomycetes and fungi (χ^2^ = 0.727; p = 0.394), with strong correspondence between the abundance of OTUs and the estimated amount of DNA included in each control.

Prior to downstream analysis of the abundance matrix, control samples and OTUs with less than 10 reads were eliminated from both the fungal and oomycete feature tables.

### Fungal and oomycete community analysis

After the abundance matrix filtering, functional guilds of the identified taxa were characterized using FunGuild database (accessed on March, 2018)^99^. Only OTUs classified with a confidence level of “Probable” or “Highly Probable” were used. The functional guilds of the remaining OTUs, classified as “Unassigned” or “Possible”, were revised based on the literature; those that were confidently classified were added to the analysis (Supplementary Information, Table S9). The rest of the unassigned OTUs and those assigned with low confidence were placed in the “Unclassified” category. Taxa classified as soil saprotrophs, plant saprotrophs, litter saprotrophs or wood saprotrophs were grouped in a unique *Soil-Plant Saprotroph* category. Those taxa that were confidently classified in more than one guild were assigned to the class >*1 Guild*, except in the case of taxa that were confidently assigned to four or more guilds, including pathogen and saprotroph guilds; these were classified in the *Opportunistic Pathogenic Species* class.

To analyze the diversity of oomycetes and fungi, abundance matrices were processed using QIIME2 version 2017.12.1^100^. Rarefaction curves, Good’s coverage, Shannon H’, OTUs abundance and Pielou evenness vector and the Bray-Curtis distance matrix were calculated using the plugin Diversity 2017.12.0^101^. The most abundant and frequent OTUs, presented in at least 50% of the samples, were selected and classified as core features using the Feature-table plugin^102^. Afterwards, the 10 most-abundant OTUs for fungi and oomycetes were selected among them and classified as the core biome (Supplementary Information Table S10). The relative abundance of the top 10 core features with respect to the number of paired sequences was calculated separately for each dataset (oomycetes and fungi). Rarefaction curves (Supplementary Information, Fig. S1) were evaluated and Good’s Coverage was calculated to estimate the adequate depth of sampling in the abundance matrices, the abundance matrices being rarefied with an even sampling depth for each dataset, with 10 iterations to avoid bias due to uneven sequencing depth. The Shannon (H’) diversity index, OTUs diversity and Evenness vector indices were calculated to study the diversity of the fungal and oomycete communities in each sample and the Bray-Curtis dissimilarity matrix was calculated; the β-diversity was analyzed to find the differences in community composition among the study zones.

### Statistical methods

All the statistical analyses were performed in the environment R Studio V 1.0.143 (RStudio Inc. 2015. Boston, MA, USA) running under R 3.4.0 (R Foundation for Statistical Computing, 2014. Vienna, Austria).

Prior to the analysis of differences of mean defoliation between zones, and for taxon abundance among defoliation level or study zones, the normality of variable residues was checked through the normal Q-Q graph and Shapiro-Wilk test. If the data did not satisfy the assumptions of homoscedasticity and normality, the logarithmic transformation was used and then, normality checked again. For normally-distributed variables, the differences were compared using ANOVA followed by a post hoc Bonferroni mean differences test. The Kruskal-Wallis rank sum test with the Bonferroni correction was used instead ANOVA when variables did not show normal distribution. Student-t Test was used for comparisons between defoliation levels.

A correlation matrix was calculated to analyse the relationships between those functional guilds present in five or more samples of all zones, thus excluding the *Lichen Parasite* and *Endophyte* guilds. Unclassified features were excluded from the correlation matrix to avoid artifacts in the analysis. The correlation analysis was used also to evaluate the relationships between the key taxa resulting from the core biome analysis. The Pearson or Spearman correlation test was used, depending on whether the two variables in each pair were distributed normally or not. Null hypotheses were rejected in all cases when p ≤ 0.05.

Alpha-diversity indices were subjected to the Kruskall-Wallis rank sum test with the Bonferroni correction, to compare the diversity levels of fungal and oomycete communities in the distinct study zones and according to the mean defoliation class of the plots. The Bray-Curtis dissimilarity matrix was used in non-metric multidimensional scaling analysis (NMDS, *vegan* package^103^, together with Analysis of Similarity (ANOSIM, 999 permutations), to determine the influence of the study location on the composition of the fungal and oomycete communities.

Other packages used for data analysis and representation were *dunn.test*, *nortest*, *ggplot2*, *ggpurb*, and *devtools* (https://CRAN.R-project.org/).

## Supplementary information

Supplementary information file
