# Supplementary material for: Assessment of functional and structural changes of soil fungal and oomycete communities in holm oak declined dehesas through metabarcoding analysis
Source: Sci Rep. 2019 Mar 29;9:5315. doi: 10.1038/s41598-019-41804-y (PMC6441054; doi:10.1038/s41598-019-41804-y)
Supplement: Supplementary file 1 — Supplementary information file [file 41598_2019_41804_MOESM1_ESM.pdf]

Assessment of functional and structural changes of soil fungal and oomycete communities in holm oak declined *dehesas* through metabarcoding analysis.

Francisco J. Ruiz Gómez, Rafael M. Navarro Cerrillo, Alejandro Pérez-de-Luque, Wolfgang Oβwald, Andrea Vannini, Carmen Morales-Rodríguez.

SUPPLEMENTARY INFORMATION

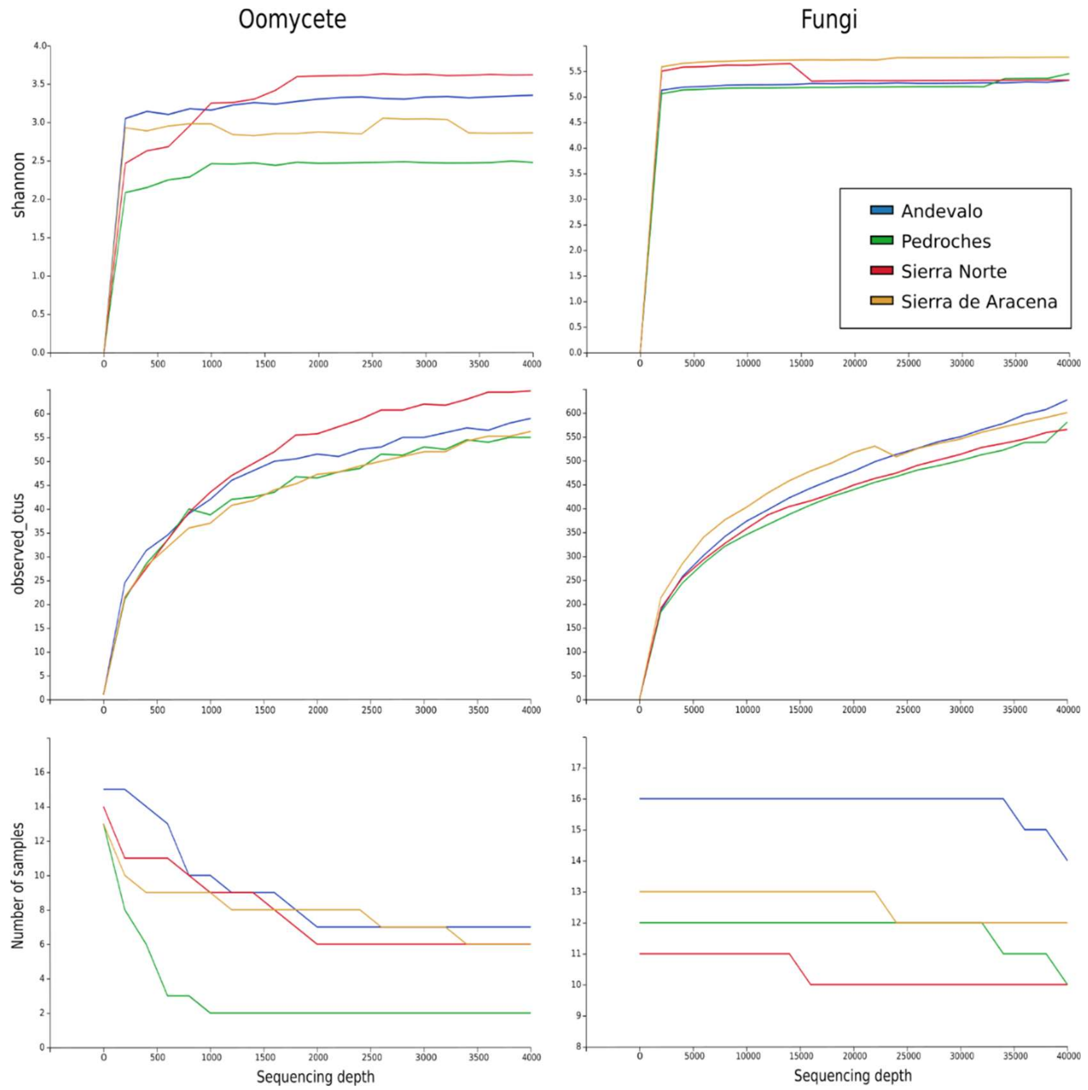

Fig. S1: Rarefaction analysis of oomycete (left) and fungi (right) raw datasets of soil total DNA library, from soil samples of Quercus ilex dehesas of the Andalusian Network for Damage Monitoring in Forest Ecosystems

Table S1: Number of OTUs identified for each class (fungus) or genus (oomycetes) (see Fig. 1 for relative abundance). UI: Unidentified taxon at the selected level (class).

Class (Fungi)	#OTUs
c__Agaricomycetes	565
c__Sordariomycetes	510
UI Ascomycota	402
c__Eurotiomycetes	307
c__Dothideomycetes	293
c__Glomeromycetes	153
c__Leotiomycetes	122
c__Pezizomycetes	88
UI Basidiomycota	84
c__Mortierellomycetes	75
c__Tremellomycetes	45
c__Chytridiomycetes	35
UI Chytridiomycota	24
c__Saccharomycetes	12
c__Microbotryomycetes	10
UI Zygomycota	9
c__Lecanoromycetes	7
c__Geoglossomycetes	6
c__Orbiliomycetes	5
o__Umbelopsidales	5
g__Pseudosigmoidea	4
c__Arthoniomycetes	2
c__Basidiobolomycetes	2
c__Cystobasidiomycetes	2
c__Entorrhizomycetes	2
c__Geminibasidiomycetes	2
c__Kickxellomycotina	2
g__Calcarisporiella	2
g__Knufia	2
UI Blastocladiomycota	2
c__Tritirachiomycetes	1
c__Wallemiomycetes	1
g__Leptodiscella	1
g__Subulispora	1
o__Mucorales	1
o__Zoopagales	1

Genus (Oomycetes)	#OTUs
g__Pythium	84
g__Phytophthora	48
g__Saprolegnia	8
g__Lagena	7
g__Aphanomyces	6
g__Achlya	3
g__Myzocytiopsis	3
g__Elongisporangium	3
g__Lagenidium	3
g__Leptolegnia	2
g__Peronospora	2
g__Apodachlya	2
g__Dictyuchus	1
g__Halophytophthora	1
s__Paralagenidium karlingii	1
g__Plectospira	1
g__Phytopythium	1
g__Elongisporangium	1
g__Phytophthora	1

Table S2: Plots selected for the study among points in *Quercus ilex* dehesas of the Andalusian Network for Damage Monitoring in Forest Ecosystems, and their main characteristics. X, Y coordinates in ETRS89 / UTM Zone 30

Plot_code	UTMX	UTMY	Zone	Prov.	Defoliation. (%)	Defoliation class	Root rot presence	*Q. ilex* (%)	Annual Preciptation. (mm)	Evapotranspiration (mm)	Annual temperature. T (ºC)	Elevation. (m.a.s.l.)
CO1004	313548	4276464	Pedr	Cordoba	35.2	C2	Yes	100%	524.17	1481.88	15.94	487
CO1013	330568	4271091	Pedr	Cordoba	30.6	C2	Yes	100%	546.29	1509.15	16.16	494
CO1023	339741	4264392	Pedr	Cordoba	43.5	C2	Yes	100%	527.86	1472.18	16.11	534
CO1029	301451	4250271	Pedr	Cordoba	25.0	C2	Yes	100%	497.68	1453.79	15.51	594
CO1055	327694	4238224	Pedr	Cordoba	26.5	C2	Yes	100%	584.06	1433.67	15.47	643
CO1060	367208	4244448	Pedr	Cordoba	22.1	C1	Yes	100%	640.99	1409.91	15.51	648
CO1101	347254	4217003	Pedr	Cordoba	25.1	C2	No	100%	591.68	1538.49	17.06	243
CO1121	294529	4192414	S.Nor	Cordoba	19.4	C1	Yes	92%	677.90	1538.55	16.91	208
HU1001	159238	4227729	Arac	Huelva	56.9	C2	Yes	85%	767.31	1452.90	16.29	344
HU1007	130122	4206909	Arac	Huelva	23.3	C1	Yes	100%	693.05	1458.21	16.94	199
HU1025	202588	4210264	Arac	Huelva	13.3	C1	Yes	100%	791.76	1363.70	15.80	581
HU1027	124740	4189836	Andv	Huelva	26.0	C2	Yes	100%	677.72	1521.43	17.10	209
HU1028	132652	4191150	Andv	Huelva	37.1	C2	No	100%	751.12	1423.43	16.66	298
HU1040	110198	4179458	Andv	Huelva	21.9	C1	Yes	100%	657.76	1545.53	17.62	154
HU1052	205011	4194513	Arac	Huelva	20.0	C1	Yes	100%	687.37	1438.48	17.13	330
HU1060	143033	4176569	Andv	Huelva	22.9	C1	Yes	96%	708.56	1476.12	17.39	171
HU1069	214215	4187820	Arac	Huelva	25.8	C2	Yes	100%	725.28	1414.34	17.14	299
HU1082	113946	4155755	Andv	Huelva	18.1	C1	Yes	100%	568.35	1499.95	17.80	144
HU1094	123107	4149092	Andv	Huelva	20.4	C1	Yes	100%	588.43	1463.71	18.03	106
HU1096	138927	4151764	Andv	Huelva	41.9	C2	Yes	100%	617.99	1396.96	17.95	116
PULIDOS	115904	4171666	Andv	Huelva	18.1	C1	Yes	100%	596.59	1515.93	17.67	148
SE1005	257837	4219069	S.Nor	Sevilla	14.8	C1	Yes	100%	706.77	1360.79	15.16	663
SE1024	253659	4194150	S.Nor	Sevilla	25.2	C2	Yes	100%	740.25	1451.89	16.20	433
SE1040	270746	4188715	S.Nor	Sevilla	18.5	C1	No	100%	765.73	1424.86	16.22	380
SE1048	240366	4175736	S.Nor	Sevilla	21.9	C1	Yes	100%	684.96	1483.04	16.99	283
SE1050	256188	4178282	S.Nor	Sevilla	21.7	C1	Yes	92%	665.11	1472.05	17.35	170

Average defoliation by zone ± SE*:* Andv: 25.8±3.2 %; Arac: 27.8±7.5 %; Pedr: 29.7±2.8 % S.Nor: 20.23±1.4 %

Table S3: Control mock communities' composition. Oo=: Oomycete control with the same concentration of DNA for each specie (ng DNA). Oo≠: Oomycete control with different concentration of DNA for each specie (ng DNA). F=: Fungal control community with the same concentration of DNA for each specie (ng DNA). F≠: Fungal control community with different concentration for each specie (ng DNA).

Oomycete controls

Strain	Name	Oo=	Oo≠
SYLV	*Pytium sylvaticum*	7.7	10
CAM124	*Phytophthora cambivora*	7.7	2
CIN10	*Phytophthora cinnamomi*	7.7	5
B1IPq	*Phytophthora quercina*	7.7	5
UND22	*Elongisporangium undulatum*	7.7	1
GON36	*Phytophthora gonapodyides*	7.7	10
1eC	*Elongisporangium anandrum*	7.7	2
CAC34	*hytophthora cactorum*	7.7	10
CIT55	*Phytophthora plurivora*	7.7	20
4BO	*Pphytophthora cithophthora*	7.7	5
MEG16	*Phytophthora megasperma*	7.7	10
AH1/2-5	*Phytophthora cryptogea*	7.7	10
PSEU1	*Phytophthora pseudosyringae*	7.7	10
		100.1	100

Fungi controls

Strain	Name	F≠	F=
T20	*Tricoderma asper*	12.5	8.3
T21	*Tricoderma virens*	5	8.3
B1.31	*Mortierella elongata*	10	8.3
F.oxy	*Fusarium oxysporum*	7.5	8.3
TA	*Fusarium ad*	2.5	8.3
VDC3574	*Vericilium dalie*	10	8.3
VTC3554	*Verticillium tricorpus*	2.5	
		50.0	50.0

Table S4: Barcodelabel tagged primers ITS 1F/ ITS 2 (Fungal dataset)

ID	Plot_code	Barcode_primer_ITS_1F	Barcode_primer_ITS_2
1-1	CO1121	CTACGGCTGATTCTYGGTCATTTAGAGGAAGTAA	GAATGTAGCHRCGTTCTTCATCGDTGC
1-2	CO1013	CGCCTGCGCTTTCTYGGTCATTTAGAGGAAGTAA	ATAAACTGCHRCGTTCTTCATCGDTGC
1-3	CO1101	CACCTGATGATCTYGGTCATTTAGAGGAAGTAA	GTTACCGAGCHRCGTTCTTCATCGDTGC
1-4	CO1023	CAAGAGATAACTYGGTCATTTAGAGGAAGTAA	GCTAGGCACGCHRCGTTCTTCATCGDTGC
1-5	CO1055	TGTAGTGTTCTYGGTCATTTAGAGGAAGTAA	ATGCTGGAATGCHRCGTTCTTCATCGDTGC
1-6	CO1013	TTCTTCCACTYGGTCATTTAGAGGAAGTAA	CATAATTCAACGCHRCGTTCTTCATCGDTGC
1-8	CO1101	CTACCATATAACTYGGTCATTTAGAGGAAGTAA	AGGTAAGCGCHRCGTTCTTCATCGDTGC
2-1	CO1029	TCAACATGTACTCTYGGTCATTTAGAGGAAGTAA	GCGATGCGCHRCGTTCTTCATCGDTGC
2-2	CO1060	CGAATACGAACCTYGGTCATTTAGAGGAAGTAA	AGGAGACGGCHRCGTTCTTCATCGDTGC
2-3	CO1004	GACCAGAGACCTYGGTCATTTAGAGGAAGTAA	CCTGCCAGAGCHRCGTTCTTCATCGDTGC
2-4	CO1055	TGCCATTGGCTYGGTCATTTAGAGGAAGTAA	GTAGGTTAGAGCHRCGTTCTTCATCGDTGC
2-5	CO1121	GAACAGTCTYGGTCATTTAGAGGAAGTAA	CTGAGGCGGATAGCHRCGTTCTTCATCGDTGC
2-6	CO1060	GAATGCCAATACCTYGGTCATTTAGAGGAAGTAA	AGAATAAGCHRCGTTCTTCATCGDTGC
2-7	CO1029	GGATTTAAAGACTYGGTCATTTAGAGGAAGTAA	ATAACGGTGCHRCGTTCTTCATCGDTGC
2-8	CO1004	ACCGCCAAGACTYGGTCATTTAGAGGAAGTAA	TTGTAGCGCGCHRCGTTCTTCATCGDTGC
3-1	SE1005	CCGGACACTCTYGGTCATTTAGAGGAAGTAA	TGCGTGCTCCGCHRCGTTCTTCATCGDTGC
3-2	SE1050	TGATTTGGCTYGGTCATTTAGAGGAAGTAA	AAGACGGAACTGCHRCGTTCTTCATCGDTGC
3-3	SE1005	TTGGTAGCTYGGTCATTTAGAGGAAGTAA	CTTTCAGTAAGGGCHRCGTTCTTCATCGDTGC
3-4	SE1024	AGTATATCCTACCTYGGTCATTTAGAGGAAGTAA	CAGCCTAGCHRCGTTCTTCATCGDTGC
3-5	SE1040	CTCACTATGATCTYGGTCATTTAGAGGAAGTAA	ATCTGCCAGCHRCGTTCTTCATCGDTGC
3-6	SE1024	GCCGCCTCTACTYGGTCATTTAGAGGAAGTAA	TTTCTACTTGCHRCGTTCTTCATCGDTGC
3-7	SE1050	CTAGCTAAGCTYGGTCATTTAGAGGAAGTAA	GCAGGAGATTGCHRCGTTCTTCATCGDTGC
3-8	SE1040	TCGAGTACCTYGGTCATTTAGAGGAAGTAA	CCAATAGACATGCHRCGTTCTTCATCGDTGC
4-1	HU1007	AAGTTGACTYGGTCATTTAGAGGAAGTAA	GTTTGTGCGCCAGCHRCGTTCTTCATCGDTGC
4-2	HU1001	ATACCTCACAAACTYGGTCATTTAGAGGAAGTAA	CTGTCCTGCHRCGTTCTTCATCGDTGC
4-3	HU1052	CCACTCATTAACTYGGTCATTTAGAGGAAGTAA	GCCTGCACGCHRCGTTCTTCATCGDTGC
4-4	PULIDOS	CGAACCATACCTYGGTCATTTAGAGGAAGTAA	GTGAAGCTGGCHRCGTTCTTCATCGDTGC
4-5	SE1048	TCGGCGAGCCTYGGTCATTTAGAGGAAGTAA	GCCTTGACCAGCHRCGTTCTTCATCGDTGC
4-6	SE1050	CGACAGAGCTYGGTCATTTAGAGGAAGTAA	GAGGCGTACAAGCHRCGTTCTTCATCGDTGC
4-7	SE1048	ATCTGGTCTYGGTCATTTAGAGGAAGTAA	TTATACTTCCATGCHRCGTTCTTCATCGDTGC
4-8	SE1024	TAAAGGATAGGCTYGGTCATTTAGAGGAAGTAA	GCGTGTTCGCHRCGTTCTTCATCGDTGC
5-1	HU1069	TTAGCAAAGCCTYGGTCATTTAGAGGAAGTAA	CCGTTCCGCGCHRCGTTCTTCATCGDTGC
5-2	HU1096	GACATTGCGACCCTYGGTCATTTAGAGGAAGTAA	TAGGAACGCHRCGTTCTTCATCGDTGC
5-3	HU1007	TGGCCTGGACTYGGTCATTTAGAGGAAGTAA	GTAGTGGAATGCHRCGTTCTTCATCGDTGC
5-4	HU1082	CGTCCAGAACAACTYGGTCATTTAGAGGAAGTAA	ACTGGTAGCHRCGTTCTTCATCGDTGC
5-5	HU1007	AGACCACATTCTYGGTCATTTAGAGGAAGTAA	GGAGACCAGGCHRCGTTCTTCATCGDTGC
5-6	HU1082	CTAGTACCACTCTYGGTCATTTAGAGGAAGTAA	TCTTTAATGCHRCGTTCTTCATCGDTGC
5-7	HU1069	GGACCTCTCACTYGGTCATTTAGAGGAAGTAA	AGACGCGAAGCHRCGTTCTTCATCGDTGC
5-8	HU1094	TCGATCCGACTYGGTCATTTAGAGGAAGTAA	CGTTTCTAGAGCHRCGTTCTTCATCGDTGC
6-1	HU1027	ATATTAAGCTYGGTCATTTAGAGGAAGTAA	GGAATACGTTGGCHRCGTTCTTCATCGDTGC

6-2	HU1052	TGTTATGCTYGGTCATTTAGAGGAAGTAA	GAATCTCAAATAGCHRCGTTCTTCATCGDTGC
6-3	HU1040	GTGCGAACTAGCCTYGGTCATTTAGAGGAAGTAA	TGTTTATGCHRCGTTCTTCATCGDTGC
6-4	HU1040	TGGCACAGCTACTYGGTCATTTAGAGGAAGTAA	CGCTTGATGCHRCGTTCTTCATCGDTGC
6-5	HU1001	CCTGACATACCTYGGTCATTTAGAGGAAGTAA	GCGTCCTCGGCHRCGTTCTTCATCGDTGC
6-6	HU1052	TGGCATAGACTYGGTCATTTAGAGGAAGTAA	CGCAGACACTGCHRCGTTCTTCATCGDTGC
6-7	HU1025	GAGGCACAACTYGGTCATTTAGAGGAAGTAA	AGATAACACTGCHRCGTTCTTCATCGDTGC
6-8	HU1060	CTACACCGCTYGGTCATTTAGAGGAAGTAA	TCTACTTGTTTGCHRCGTTCTTCATCGDTGC
7-1	HU1001	TCGTGATCTYGGTCATTTAGAGGAAGTAA	CCTCTCACATGGGCHRCGTTCTTCATCGDTGC
7-2	HU1094	CGCTCGGACTYGGTCATTTAGAGGAAGTAA	TCAGACCTTCAGCHRCGTTCTTCATCGDTGC
7-3	HU1028	AGCCTATCCTTTCTYGGTCATTTAGAGGAAGTAA	GGTCAGAGCHRCGTTCTTCATCGDTGC
7-4	HU1096	ACGTGTGCTYGGTCATTTAGAGGAAGTAA	CCGCGTTCACCTGCHRCGTTCTTCATCGDTGC
7-5	HU1069	ACATATCATAGCTYGGTCATTTAGAGGAAGTAA	GTGGCTTTGCHRCGTTCTTCATCGDTGC
7-6	HU1028	AACTGGCACCCTYGGTCATTTAGAGGAAGTAA	CTCATTCCTGCHRCGTTCTTCATCGDTGC
7-7	HU1094	CGTCCTTCGCTYGGTCATTTAGAGGAAGTAA	GGCTTAATTAGCHRCGTTCTTCATCGDTGC
7-8	HU1060	GACACTGTCTYGGTCATTTAGAGGAAGTAA	ATGCACCAGCAGCHRCGTTCTTCATCGDTGC
8-1	HU1025	GCTCCGCCTYGGTCATTTAGAGGAAGTAA	CGAAACGCTATAGCHRCGTTCTTCATCGDTGC
8-2	PULIDOS	TGCGACTGTGGCCTYGGTCATTTAGAGGAAGTAA	GTCCAGCGCHRCGTTCTTCATCGDTGC
F=	Control 1	CTTTAATAGCCTYGGTCATTTAGAGGAAGTAA	GATTGAAACGCHRCGTTCTTCATCGDTGC
F=	Control 1	GGCATTAACCTYGGTCATTTAGAGGAAGTAA	CAGGCAAGCAGCHRCGTTCTTCATCGDTGC
F=	Control 1	TGCCTTCACTYGGTCATTTAGAGGAAGTAA	GGATCACTCCTGCHRCGTTCTTCATCGDTGC
F≠	Control 2	GCCTAAGCTYGGTCATTTAGAGGAAGTAA	TTAGGAACAATTGCHRCGTTCTTCATCGDTGC
F≠	Control 2	TTTGAATACCACCTYGGTCATTTAGAGGAAGTAA	AGCTCAAGCHRCGTTCTTCATCGDTGC
F≠	Control 2	TCCGTACTGCCCTYGGTCATTTAGAGGAAGTAA	CGTTTGCCGCHRCGTTCTTCATCGDTGC

Table S5: Barcodelabel tagged primers ITS 6/ ITS 7 (Oomycete dataset)

ID	Plot_code	Barcode_primer_ITS_6	Barcode_primer_ITS_7
1-1	CO1121	CTTAATCCAGGTGAAGGTGAAGTCGTAACAAGG	TCCGCCTAGCGTTCTTCATCGATGTGC
1-2	CO1013	GCCAGTTCAGTAGAAGGTGAAGTCGTAACAAGG	TGTACGAAGCGTTCTTCATCGATGTGC
1-3	CO1101	CCTAAGATGTTGAAGGTGAAGTCGTAACAAGG	TGCACTCAAGCGTTCTTCATCGATGTGC
1-4	CO1023	GTCAGAGGCAGAAGGTGAAGTCGTAACAAGG	ATGCGAGACAGCGTTCTTCATCGATGTGC
1-5	CO1055	CATACATGAAGGTGAAGTCGTAACAAGG	AAATAGGCAATAAGCGTTCTTCATCGATGTGC
1-6	CO1013	CATACGCAGAAGGTGAAGTCGTAACAAGG	GGCATCTAATAAGCGTTCTTCATCGATGTGC
1-8	CO1101	CCGAAACCGACGAAGGTGAAGTCGTAACAAGG	GCATACAGAGCGTTCTTCATCGATGTGC
2-1	CO1029	GTCCACATAATCGAAGGTGAAGTCGTAACAAGG	TAGCCGAAGCGTTCTTCATCGATGTGC
2-2	CO1060	CCAGAATTAGAGAAGGTGAAGTCGTAACAAGG	TTGGCTAGAGCGTTCTTCATCGATGTGC
2-3	CO1004	CACGTAAGTCGAAGGTGAAGTCGTAACAAGG	TGTGATCTAAGCGTTCTTCATCGATGTGC
2-4	CO1055	GACTAGCTGGAAGGTGAAGTCGTAACAAGG	ATACCAAACTAGCGTTCTTCATCGATGTGC
2-5	CO1121	ACGTTAAGGAAGGTGAAGTCGTAACAAGG	CAGATCCGCTCAGCGTTCTTCATCGATGTGC
2-6	CO1060	GTCGAGGGAAGGTGAAGTCGTAACAAGG	TAATCGTCTAATAGCGTTCTTCATCGATGTGC
2-7	CO1029	AGTTTGTAGTAAGAAGGTGAAGTCGTAACAAGG	GCGCCATAGCGTTCTTCATCGATGTGC
2-8	CO1004	ACGGAGCTACCGAAGGTGAAGTCGTAACAAGG	GCATTTGAAGCGTTCTTCATCGATGTGC
3-1	SE1005	TCTAGTGCCTACGAAGGTGAAGTCGTAACAAGG	GATCACTAGCGTTCTTCATCGATGTGC
3-2	SE1050	ATTGTGGTGGAAGGTGAAGTCGTAACAAGG	GAATGGTCAGAGCGTTCTTCATCGATGTGC
3-3	SE1005	ACGAATCAACTGAAGGTGAAGTCGTAACAAGG	CTGCGTTCAGCGTTCTTCATCGATGTGC
3-4	SE1024	GGCGACAGAAGGTGAAGTCGTAACAAGG	ACGCTATGATGTAGCGTTCTTCATCGATGTGC
3-5	SE1040	TCGATGTGGAATGAAGGTGAAGTCGTAACAAGG	CAGCTAAAGCGTTCTTCATCGATGTGC
3-6	SE1024	AGCTTGGATAGAAGGTGAAGTCGTAACAAGG	TAAGCTAACAGCGTTCTTCATCGATGTGC
3-7	SE1050	TTTAAGCGACGAAGGTGAAGTCGTAACAAGG	ACCGGCGTTAGCGTTCTTCATCGATGTGC
3-8	SE1040	TATACTTGTGAAGGTGAAGTCGTAACAAGG	CCTGACGTGAAGCGTTCTTCATCGATGTGC
4-1	HU1007	CTTCAAGGGAAGGTGAAGTCGTAACAAGG	TGCGCGGCTCAAGCGTTCTTCATCGATGTGC
4-2	HU1001	TAACAAGGAAGGTGAAGTCGTAACAAGG	AGCATTGAGATGAGCGTTCTTCATCGATGTGC
4-3	HU1052	GATTATCAGTCGAAGGTGAAGTCGTAACAAGG	AACGAGAAAGCGTTCTTCATCGATGTGC
4-4	PULIDOS	CCAACTGTCCGGAAGGTGAAGTCGTAACAAGG	AGACGGCTAGCGTTCTTCATCGATGTGC
4-5	SE1048	TAATCAGTGAAGGTGAAGTCGTAACAAGG	ATTAACTTCCAAGCGTTCTTCATCGATGTGC
4-6	SE1050	AACCAATGAGAAGGTGAAGTCGTAACAAGG	GAGAAACAATAGCGTTCTTCATCGATGTGC
4-7	SE1048	GATAAGGGAAGGTGAAGTCGTAACAAGG	AATTGACAACTGAGCGTTCTTCATCGATGTGC
4-8	SE1024	ACTACGTGAAGGTGAAGTCGTAACAAGG	TCGCGGCCTTTGAGCGTTCTTCATCGATGTGC
5-1	HU1069	CAACCGGATTAGGAAGGTGAAGTCGTAACAAGG	GACCTCTAGCGTTCTTCATCGATGTGC
5-2	HU1096	AGTAACCGAAGGAAGGTGAAGTCGTAACAAGG	TTATTAGAAGCGTTCTTCATCGATGTGC
5-3	HU1007	GTTCCTATGCGGAAGGTGAAGTCGTAACAAGG	CACACACTAGCGTTCTTCATCGATGTGC
5-4	HU1082	TTAACCTACGAAGGTGAAGTCGTAACAAGG	GCTAATCTTGAGCGTTCTTCATCGATGTGC
5-5	HU1007	CCTGCTACACGAAGGTGAAGTCGTAACAAGG	GCCGCAGAAAGCGTTCTTCATCGATGTGC
5-6	HU1082	CAAACATAGAAGGTGAAGTCGTAACAAGG	TGCGGTAGCAAAGCGTTCTTCATCGATGTGC
5-7	HU1069	ACAATAGGAAGGTGAAGTCGTAACAAGG	GAAAGCACCTATAGCGTTCTTCATCGATGTGC
5-8	HU1094	AGACGTTACAGAGAAGGTGAAGTCGTAACAAGG	CTAGCGAAGCGTTCTTCATCGATGTGC
6-1	HU1027	GAGCCATTAACGAAGGTGAAGTCGTAACAAGG	AAACTGTAAGCGTTCTTCATCGATGTGC

6-2	HU1052	CAATCCACCAGAAGGTGAAGTCGTAACAAGG	GGCATAGCAAGCGTTCTTCATCGATGTGC
6-3	HU1040	AGCTCTTCAAGAAGGTGAAGTCGTAACAAGG	GGAGATTGCAGCGTTCTTCATCGATGTGC
6-4	HU1040	GCAACGGTAGAAGGTGAAGTCGTAACAAGG	ACTGTACAAAAGCGTTCTTCATCGATGTGC
6-5	HU1001	AGAATTGGAAGGTGAAGTCGTAACAAGG	GGCCAAGCTATTAGCGTTCTTCATCGATGTGC
6-6	HU1052	CTACAGCCTACAGAAGGTGAAGTCGTAACAAGG	TCCTATAAGCGTTCTTCATCGATGTGC
6-7	HU1025	ACCTCAAATGAAGGTGAAGTCGTAACAAGG	TTAATTTCTAAGCGTTCTTCATCGATGTGC
6-8	HU1060	ACAGACGACCAGAAGGTGAAGTCGTAACAAGG	TTAAATACAGCGTTCTTCATCGATGTGC
7-1	HU1001	GCGGCCAGGCGAAGGTGAAGTCGTAACAAGG	CCATTCAGGAGCGTTCTTCATCGATGTGC
7-2	HU1094	ATCCTGCCGAAGGTGAAGTCGTAACAAGG	TATTCTTGACTAGCGTTCTTCATCGATGTGC
7-3	HU1028	TACTAAACGAAGGTGAAGTCGTAACAAGG	AAGCATGAAGGAGCGTTCTTCATCGATGTGC
7-4	HU1096	AGCAAGGGAAGGTGAAGTCGTAACAAGG	GCTGCCGCAATAAGCGTTCTTCATCGATGTGC
7-5	HU1069	CCAGGACTGAAGGTGAAGTCGTAACAAGG	TCTACAACATAAGCGTTCTTCATCGATGTGC
7-6	HU1028	AAGGCGAGAAGGTGAAGTCGTAACAAGG	GAGGTTATAGGCAGCGTTCTTCATCGATGTGC
7-7	HU1094	CAAAGCTTAATAGAAGGTGAAGTCGTAACAAGG	TAGGACCAGCGTTCTTCATCGATGTGC
7-8	HU1060	GGACGAAGAAGGTGAAGTCGTAACAAGG	TACTCCGATACTAGCGTTCTTCATCGATGTGC
8-1	HU1025	GTCCAAAGCGGAAGGTGAAGTCGTAACAAGG	ATATATCAAAGCGTTCTTCATCGATGTGC
8-2	PULIDOS	TCGTTTAGGTAAGAAGGTGAAGTCGTAACAAGG	ATGGACTAGCGTTCTTCATCGATGTGC
Oo=	Control 3	GAGTAAACAGAAGGTGAAGTCGTAACAAGG	TTTGTTCAATAGCGTTCTTCATCGATGTGC
Oo=	Control 3	GGAGCCTAGAAGGTGAAGTCGTAACAAGG	CCTATGTTATTAGCGTTCTTCATCGATGTGC
Oo=	Control 3	GCAAATAGAAGGTGAAGTCGTAACAAGG	ACTCCGTAGACAAGCGTTCTTCATCGATGTGC
Oo≠	Control 4	GCAAGCTCTAGAAGGTGAAGTCGTAACAAGG	TATATGTGTAGCGTTCTTCATCGATGTGC
Oo≠	Control 4	GCAAGCTCTAGAAGGTGAAGTCGTAACAAGG	ACTAAAGAGCGTTCTTCATCGATGTGC
Oo≠	Control 4	CACATTATAGGAAGGTGAAGTCGTAACAAGG	GATAGTAAGAGCGTTCTTCATCGATGTGC

Table S6: Illumina de novo sequencing results of soil total DNA library, from soil samples of Quercus ilex dehesas of the Andalusian Network for Damage Monitoring in Forest Ecosystems. A: Statistics of the sequencing project referred to the whole raw data. B: Resume of debarcoded sequences to the fungi and oomycete dataset after debarcoding and grouping of ITS1F/2 and ITS6/7 identified tagged primers.

A

Type	%Q30	MeanQ	#Cluster
Illumina MiSeq 2x300	84.34%	33.52	14 732 937

B

		Debarcoded Seqs.	
**Features**	**Project**	**ITS1F/2**	**ITS6/7**
Forward sequence only	3 639 702	2 375 465	1 264 259
Reverse sequence only	1 805 367	1 456 828	348 535
Paired sequences	8 041 574	6 769 397	1 272 177
Mean freq. Per sample	101 366.60	90 593.20	9025.70
Sign. OTU's	4193	3912	281
Mean OTU freq	1378.00	1343.10	1862.90

Table S7: Parameters of sequence processing in CLC Genomics Workbench 3.6.5.

Input	Value
Trimming limit	0.5
Ambiguous bases threshold	0
Minimum read size	100 bp
Maximum read size	500 bp
Overlapping mismatch	1
Overlapping gap cost	3
Minimum score	50

Table S8 LCA Parameters of taxonomy processing in MEGAN 6.10.8

Input	Value
Minimum score threshold	170
Minimum sequence similarity	99%
Minimum LCA percent	50%

Table S9 References of the revised functional guilds (page 1 of 6)

OTU ID	Taxon	Guild	Citation/Source
fungi_OTU_1017	Peniophora	Plant Pathogen	doi.org/10.1016/S0953-7562(96)80118-3
fungi_OTU_1027	Thelephora	Ectomycorrhizal	ISBN 978-0-85198-786-6
fungi_OTU_1033	Gelasinospora	Soil-Plant Saprotroph	Lundqvist N, (1972). Nordic Sordariaceae S. Lat. Symbolae Botanicae Upsaliensis, 20:1-374
fungi_OTU_104	Trichophaea	Soil-Plant Saprotroph	Kanouse B. (1958). Some Speccies of the Genus Trichophaea. Mycologia, 50:121-140
fungi_OTU_1065	Thelephora	Ectomycorrhizal	ISBN 978-0-85198-786-6
fungi_OTU_1080	Peniophora	Plant Pathogen	doi.org/10.1016/S0953-7562(96)80118-3
fungi_OTU_1192	Sordaria	Dung Saprotroph	Guarro J, Arx JA. (1987) The Ascomycete genus Sordaria. Persoonia 13:301-313
fungi_OTU_1205	Gelasinospora	Soil-Plant Saprotroph	Lundqvist N, (1972). Nordic Sordariaceae S. Lat. Symbolae Botanicae Upsaliensis, 20:1-374
fungi_OTU_1245	Hawksworthiomyces	Soil-Plant Saprotroph	10.1016/j.funbio.2016.07.004
fungi_OTU_1342	Coprinellus	Soil-Plant Saprotroph	10.3852/11-149
fungi_OTU_1415	Ppseudareulia	Soil-Plant Saprotroph	http://www.indexfungorum.org
fungi_OTU_1429	Preussia	Dung Saprotroph	Lumbsch, H. T. and S.M. Huhndorf (ed.) 2007. Outline of Ascomycota – 2007. Myconet 13: 1 - 58.
fungi_OTU_1431	Peniophora	Plant Pathogen	doi.org/10.1016/S0953-7562(96)80118-3
fungi_OTU_1434	Preussia	Dung Saprotroph	Lumbsch, H. T. and S.M. Huhndorf (ed.) 2007. Outline of Ascomycota – 2007. Myconet 13: 1 - 58.
fungi_OTU_152	Thelephora	Ectomycorrhizal	ISBN 978-0-85198-786-6
fungi_OTU_1535	Laccaria	Ectomycorrhizal	doi.org/10.1016/0167-8809(90)90043-D
fungi_OTU_1578	Scedosporium	Animal Pathogen	doi.org/10.4067/S0716-10182012000300014
fungi_OTU_160	Ramophialophora	Soil-Plant Saprotroph	doi.org/10.3852/09-128
fungi_OTU_1604	Verrucaria	Lichenized	doi.org/10.1016/j.mycres.2007.08.010
fungi_OTU_1679	Microdochium	Plant Pathogen	doi.org/10.1139/b72-240
fungi_OTU_1721	Cephalotrichum	Soil-Plant Saprotroph	doi.org/10.1016/j.simyco.2017.09.001
fungi_OTU_1737	Pseudophialophora	>1 guild	doi.org/10.3852/13-306
fungi_OTU_1780	Thelephora	Ectomycorrhizal	ISBN 978-0-85198-786-6
fungi_OTU_1811	Oidiodendron	Soil-Plant Saprotroph	doi.org/10.3114/sim.53.1.83
fungi_OTU_1815	Apiosordaria	Dung saprotroph	Krug, J.C., Udagawa, S., Jeng, R.S. (1983). The genus Apiiosordaria. Mycotaxon 17:533-549
fungi_OTU_1846	Peniophora	Plant Pathogen	doi.org/10.1016/S0953-7562(96)80118-3
fungi_OTU_1875	Oidiodendron	Soil-Plant Saprotroph	doi.org/10.3114/sim.53.1.83
fungi_OTU_1881	Calyptella	Plant Pathogen	doi.org/10.1111/j.1365-3059.1983.tb01306.x
fungi_OTU_197	Microdochium	Plant Pathogen	doi.org/10.1139/b72-240
fungi_OTU_1979	Preussia	Dung Saprotroph	Lumbsch, H. T. and S.M. Huhndorf (ed.) 2007. Outline of Ascomycota – 2007. Myconet 13: 1 - 58.

Table S9 References of the revised functional guilds (page 2 of 6)

OTU ID	Taxon	Guild	Citation/Source
fungi_OTU_1994	Cryptotrichosporon	Soil-Plant Saprotroph	doi.org/10.1111/j.1567-1364.2006.00164.x
fungi_OTU_2030	Heteroconium	Endophyte	doi.org/10.1016/j.femsle.2005.08.039
fungi_OTU_2156	Rutstroemia	Soil-Plant Saprotroph	Lumbsch TH, Huhndorf SM. (2007). Outline of Ascomycota – 2007. Myconet. Chicago, USA: The Field Museum, Department of Botany. 13: 1–58.
fungi_OTU_2167	Oidiodendron	Soil-Plant Saprotroph	doi.org/10.3114/sim.53.1.83
fungi_OTU_2197	Sordaria	Dung Saprotroph	Guarro J, Arx JA. (1987) The Ascomycete genus Sordaria. Persoonia 13:301-313
fungi_OTU_2207	Monosporascus	Plant Pathogen	Lobo Ruano et al., 1990 Bol. San Veg y Plagas 16:701-707
fungi_OTU_2210	Cadophora	Plant Pathogen	doi.org/10.1016/j.funbio.2014.11.002
fungi_OTU_2218	Rutstroemia	Soil-Plant Saprotroph	Lumbsch TH, Huhndorf SM. (2007). "Outline of Ascomycota – 2007". Myconet. Chicago, USA: The Field Museum, Department of Botany. 13: 1–58.
fungi_OTU_2231	Pseudophialophora	>1 guild	doi.org/10.3852/13-306
fungi_OTU_2245	Scutellinia	Soil-Plant Saprotroph	Schumacher, T. (1990). The genus Scutellinia (Pyronemataceae). Opera Botanica 101: 1-107
fungi_OTU_2318	Pseudogymnoascus	Soil-Plant Saprotroph	doi.org/10.1007/bf02461670.
fungi_OTU_2340	Pseudophialophora	>1 guild	doi.org/10.3852/13-306
fungi_OTU_2375	Cadophora	Plant Pathogen	doi.org/10.1016/j.funbio.2014.11.002
fungi_OTU_2389	Hawksworthiomyces	Soil-Plant Saprotroph	doi.org/10.1016/j.funbio.2016.07.004
fungi_OTU_2407	Delicatula	Soil-Plant Saprotroph	Velenovský J. (1947). Novitates mycologicae novissimae. Opera botanica Čechica (in Latin). 4. Prague: Societas Botanica Čechoslovaca. p. 33
fungi_OTU_2439	Spizellomyces	Soil-Plant Saprotroph	doi.org/10.2307/3792840
fungi_OTU_2464	Syncephalis	Fungal Parasite	doi.org/10.2307/3756922
fungi_OTU_2498	Tetracladium	Soil-Plant Saprotroph	doi.org/10.11646/phytotaxa.338.3.5
fungi_OTU_253	Oidiodendron	Soil-Plant Saprotroph	doi.org/10.3114/sim.53.1.83
fungi_OTU_2531	Ppseudareulia	Soil-Plant Saprotroph	http://www.indexfungorum.org
fungi_OTU_2533	Oidiodendron	Soil-Plant Saprotroph	doi.org/10.3114/sim.53.1.83
fungi_OTU_259	Preussia	Dung Saprotroph	Lumbsch, H. T. and S.M. Huhndorf (ed.) 2007. Outline of Ascomycota – 2007. Myconet 13: 1 - 58.
fungi_OTU_2590	Vermispora	Animal Pathogen	Burghouts, T.; Gams, W. (1989). Vermispora fusarina, a new hyphomycete parasitizing cyst nematodes. Mem. N. Y. bot. Gdn 49: 57-61.
fungi_OTU_2668	Oidiodendron	Soil-Plant Saprotroph	doi.org/10.3114/sim.53.1.83
fungi_OTU_275	Preussia	Dung Saprotroph	Lumbsch, H. T. and S.M. Huhndorf (ed.) 2007. Outline of Ascomycota – 2007. Myconet 13: 1 - 58.
fungi_OTU_2758	Monacrosporium	Animal Pathogen	doi.org/10.1128/AEM.01390-13.
fungi_OTU_2830	Oidiodendron	Soil-Plant Saprotroph	doi.org/10.3114/sim.53.1.83
fungi_OTU_2840	Hohenbuehelia	Animal Pathogen	doi.org/10.1139/b77-345

Table S9 References of the revised functional guilds (page 3 of 6)

OTU ID	Taxon	Guild	Citation/Source
fungi_OTU_289	Trichophaea	Soil-Plant Saprotroph	Kanouse B. (1958). Some Speccies of the Genus Trichophaea. Mycologia, 50:121-140
fungi_OTU_2927	Peniophora	Plant Pathogen	doi.org/10.1016/S0953-7562(96)80118-3
fungi_OTU_2974	Sordaria	Dung Saprotroph	Guarro J, Arx JA. (1987) The Ascomycete genus Sordaria. Persoonia 13:301-313
fungi_OTU_2979	Preussia	Dung Saprotroph	Lumbsch, H. T. and S.M. Huhndorf (ed.) 2007. Outline of Ascomycota – 2007. Myconet 13: 1 - 58.
fungi_OTU_2986	Cryptotrichosporon	Soil-Plant Saprotroph	doi.org/10.1111/j.1567-1364.2006.00164.x
fungi_OTU_3062	Monosporascus	Plant Pathogen	Lobo Ruano et al., 1990 Bol. San Veg y Plagas 16:701-707
fungi_OTU_3096	Tetracladium	Soil-Plant Saprotroph	doi.org/10.11646/phytotaxa.338.3.5
fungi_OTU_3101	Preussia	Dung Saprotroph	Lumbsch, H. T. and S.M. Huhndorf (ed.) 2007. Outline of Ascomycota – 2007. Myconet 13: 1 - 58.
fungi_OTU_3112	Ramicandelaber	Soil-Plant Saprotroph	doi.org/10.1017/S0953756204000930
fungi_OTU_3212	Coprinellus	Soil-Plant Saprotroph	doi.org/10.3852/11-149
fungi_OTU_323	Distoseptispora	Soil-Plant Saprotroph	doi.org/10.1007/s13225-016-0373-x
fungi_OTU_3291	Ramicandelaber	Soil-Plant Saprotroph	doi.org/10.1017/S0953756204000930
fungi_OTU_3327	Peniophora	Plant Pathogen	doi.org/10.1016/S0953-7562(96)80118-3
fungi_OTU_3332	Pseudophialophora	>1 guild	doi.org/10.3852/13-306
fungi_OTU_3434	Rhexodenticula	Soil-Plant Saprotroph	http://www.indexfungorum.org
fungi_OTU_3524	Coprinellus	Soil-Plant Saprotroph	doi.org/10.3852/11-149
fungi_OTU_3556	Monosporascus	Plant Pathogen	Lobo Ruano et al., 1990 Bol. San Veg y Plagas 16:701-707
fungi_OTU_3563	Cercophora	Soil-Plant Saprotroph	doi.org/10.3852/11-005
fungi_OTU_3687	Peniophora	Plant Pathogen	doi.org/10.1016/S0953-7562(96)80118-3
fungi_OTU_3698	Oidiodendron	Soil-Plant Saprotroph	doi.org/10.3114/sim.53.1.83
fungi_OTU_3759	Peniophora	Plant Pathogen	doi.org/10.1016/S0953-7562(96)80118-3
fungi_OTU_3774	Sordaria	Dung Saprotroph	Guarro J, Arx JA. (1987) The Ascomycete genus Sordaria. Persoonia 13:301-313
fungi_OTU_387	Gymnoascus	Soil-Plant Saprotroph	Lumbsch TH, Huhndorf SM (December 2007). "Outline of Ascomycota – 2007". Myconet. Chicago, USA: The Field Museum, Department of Botany. 13: 1–58.
fungi_OTU_3902	Parasola	Soil-Plant Saprotroph	doi.org/10.1080/00275514.2017.1386526
fungi_OTU_3903	Microascus	Soil-Plant Saprotroph	doi.org/10.2323/jgam.8.39
fungi_OTU_3979	Peniophora	Plant Pathogen	doi.org/10.1016/S0953-7562(96)80118-3
fungi_OTU_437	Trichophaea	Soil-Plant Saprotroph	Kanouse B. (1958). Some Speccies of the Genus Trichophaea. Mycologia, 50:121-140
fungi_OTU_443	Cadophora	Plant Pathogen	doi.org/10.1016/j.funbio.2014.11.002
fungi_OTU_45	Laccaria	Ectomycorrhizal	doi.org/10.1016/0167-8809(90)90043-D
fungi_OTU_584	Sordaria	Dung Saprotroph	Guarro J, Arx JA. (1987) The Ascomycete genus Sordaria. Persoonia 13:301-313

Table S9 References of the revised functional guilds (page 4 of 6)

OTU ID	Taxon	Guild	Citation/Source
fungi_OTU_59	Laccaria	Ectomycorrhizal	doi.org/10.1016/0167-8809(90)90043-D
fungi_OTU_598	Tremellodendron	>1 guild	Weiss M, Selosse MA, Rexer KH, Urban A, Oberwinkler F (2004). "Sebacinales: a hitherto overlooked cosm of heterobasidiomycetes with a broad mycorrhizal potential". Mycological Research. 108 (Pt 9): 1003–10. doi:10.1017/S0953756204000772. PMID 15506013.
fungi_OTU_602	Cadophora	Plant Pathogen	doi.org/10.1016/j.funbio.2014.11.002
fungi_OTU_618	Cutaneotrichosporon	Animal Pathogen	doi.org/10.1007/s00203-016-1254-0
fungi_OTU_621	Preussia	Dung Saprotroph	Lumbsch, H. T. and S.M. Huhndorf (ed.) 2007. Outline of Ascomycota – 2007. Myconet 13: 1 - 58.
fungi_OTU_674	Monosporascus	Plant Pathogen	Lobo Ruano et al., 1990 Bol. San Veg y Plagas 16:701-707
fungi_OTU_685	Thelephora	Ectomycorrhizal	ISBN 978-0-85198-786-6
fungi_OTU_70	Ppseudareulia	Soil-Plant Saprotroph	http://www.indexfungorum.org
fungi_OTU_71	Thelephora	Ectomycorrhizal	ISBN 978-0-85198-786-6
fungi_OTU_722	Cercophora	Soil-Plant Saprotroph	doi.org/10.3852/11-005
fungi_OTU_744	Cadophora	Plant Pathogen	doi.org/10.1016/j.funbio.2014.11.002
fungi_OTU_75	Sordaria	Dung Saprotroph	Guarro J, Arx JA. (1987) The Ascomycete genus Sordaria. Persoonia 13:301-313
fungi_OTU_79	Wilcoxinia	Ectomycorrhizal	Nakas, J.P., Hagedorn, C. (1990). Biotechnology of Plan-Microbe interactions. McGrawn-Hill. ISBN 978-0-07-045867-3
fungi_OTU_816	Peniophora	Plant Pathogen	doi.org/10.1016/S0953-7562(96)80118-3
fungi_OTU_839	Sordaria	Dung Saprotroph	Guarro J, Arx JA. (1987) The Ascomycete genus Sordaria. Persoonia 13:301-313
fungi_OTU_846	Peniophora	Plant Pathogen	doi.org/10.1016/S0953-7562(96)80118-3
fungi_OTU_862	Cadophora	Plant Pathogen	doi.org/10.1016/j.funbio.2014.11.002
fungi_OTU_863	Monosporascus	Plant Pathogen	Lobo Ruano et al., 1990 Bol. San Veg y Plagas 16:701-707
fungi_OTU_879	Monosporascus	Plant Pathogen	Lobo Ruano et al., 1990 Bol. San Veg y Plagas 16:701-707
fungi_OTU_898	Tetracladium	Soil-Plant Saprotroph	doi.org/10.11646/phytotaxa.338.3.5
fungi_OTU_904	Ppseudareulia	Soil-Plant Saprotroph	http://www.indexfungorum.org
fungi_OTU_907	Vascellum	Soil-Plant Saprotroph	doi.org/10.5943/mycosphere/4/4/11
fungi_OTU_955	Preussia	Dung Saprotroph	Lumbsch, H. T. and S.M. Huhndorf (ed.) 2007. Outline of Ascomycota – 2007. Myconet 13: 1 - 58.
oomycete_OTU_126	Myzocytiopsis	Animal Pathogen	Glocking and Beakes, 2000. A review of the taxonomy, biology and infection strategies of "biflagellate holocarpic" parasites of nematodes. Fungal Diversity 4: 1-20
oomycete_OTU_130	Lagenidium	Pathotroph-Saprotroph Oportunistic species	doi.org/10.1007/s13225-014-0298-1
oomycete_OTU_150	Apodachlya	Soil-Plant Saprotroph	doi.org/10.1007/BF00418292

Table S9 References of the revised functional guilds (page 5 of 6)

OTU ID	Taxon	Guild	Citation/Source
oomycete_OTU_156	Saprolegnia	Pathotroph-Saprotroph Oportunistic species	1) Beakes, G.W., Wood, S.E. & Burr, A.W. (1994) Features which characterize Saprolegnia isolates from salmon fish lesions – a review. In: Mueller, G.J. (Ed.) Salmon Saprolegniasis. Report to Bonneville Power Administration. Environment, Fish and Wildlife Division, Portland, Oregon. pp. 33–66. 2) van den Berg, A.H., McLaggan, D., Diéguez-Uribeondo, J. & van West, P. (2013) The impact of the water moulds Saprolegnia diclina and Saprolegnia parasitica on natural ecosystems and the aquaculture industry. Fungal Biology Reviews 27: 33–42. http://dx.doi.org/10.1016/j.fbr.2013.05.001
oomycete_OTU_171	Saprolegnia	Pathotroph-Saprotroph Oportunistic species	1) Beakes, G.W., Wood, S.E. & Burr, A.W. (1994) Features which characterize Saprolegnia isolates from salmon fish lesions – a review. In: Mueller, G.J. (Ed.) Salmon Saprolegniasis. Report to Bonneville Power Administration. Environment, Fish and Wildlife Division, Portland, Oregon. pp. 33–66. 2) van den Berg, A.H., McLaggan, D., Diéguez-Uribeondo, J. & van West, P. (2013) The impact of the water moulds Saprolegnia diclina and Saprolegnia parasitica on natural ecosystems and the aquaculture industry. Fungal Biology Reviews 27: 33–42. http://dx.doi.org/10.1016/j.fbr.2013.05.001
oomycete_OTU_197	Saprolegnia	Pathotroph-Saprotroph Oportunistic species	1) Beakes, G.W., Wood, S.E. & Burr, A.W. (1994) Features which characterize Saprolegnia isolates from salmon fish lesions – a review. In: Mueller, G.J. (Ed.) Salmon Saprolegniasis. Report to Bonneville Power Administration. Environment, Fish and Wildlife Division, Portland, Oregon. pp. 33–66. 2) van den Berg, A.H., McLaggan, D., Diéguez-Uribeondo, J. & van West, P. (2013) The impact of the water moulds Saprolegnia diclina and Saprolegnia parasitica on natural ecosystems and the aquaculture industry. Fungal Biology Reviews 27: 33–42. http://dx.doi.org/10.1016/j.fbr.2013.05.001
oomycete_OTU_211	Myzocytiopsis	Animal Pathogen	Glocking and Beakes, 2000. A review of the taxonomy, biology and infection strategies of "biflagellate holocarpic" parasites of nematodes. Fungal Diversity 4: 1-20
oomycete_OTU_213	Saprolegnia	Pathotroph-Saprotroph Oportunistic species	1) Beakes, G.W., Wood, S.E. & Burr, A.W. (1994) Features which characterize Saprolegnia isolates from salmon fish lesions – a review. In: Mueller, G.J. (Ed.) Salmon Saprolegniasis. Report to Bonneville Power Administration. Environment, Fish and Wildlife Division, Portland, Oregon. pp. 33–66. 2) van den Berg, A.H., McLaggan, D., Diéguez-Uribeondo, J. & van West, P. (2013) The impact of the water moulds Saprolegnia diclina and Saprolegnia parasitica on natural ecosystems and the aquaculture industry. Fungal Biology Reviews 27: 33–42. http://dx.doi.org/10.1016/j.fbr.2013.05.001
oomycete_OTU_247	Myzocytiopsis	Animal Pathogen	Glocking and Beakes, 2000. A review of the taxonomy, biology and infection strategies of "biflagellate holocarpic" parasites of nematodes. Fungal Diversity 4: 1-20

Table S9 References of the revised functional guilds (page 6 of 6)

OTU ID	Taxon	Guild	Citation/Source
oomycete_OTU_253	Saprolegnia	Pathotroph-Saprotroph Oportunistic species	1) Beakes, G.W., Wood, S.E. & Burr, A.W. (1994) Features which characterize Saprolegnia isolates from salmon fish lesions – a review. In: Mueller, G.J. (Ed.) Salmon Saprolegniasis. Report to Bonneville Power Administration. Environment, Fish and Wildlife Division, Portland, Oregon. pp. 33–66. 2) van den Berg, A.H., McLaggan, D., Diéguez-Uribeondo, J. & van West, P. (2013) The impact of the water moulds Saprolegnia diclina and Saprolegnia parasitica on natural ecosystems and the aquaculture industry. Fungal Biology Reviews 27: 33–42. http://dx.doi.org/10.1016/j.fbr.2013.05.001
oomycete_OTU_262	Saprolegnia	Pathotroph-Saprotroph Oportunistic species	1) Beakes, G.W., Wood, S.E. & Burr, A.W. (1994) Features which characterize Saprolegnia isolates from salmon fish lesions – a review. In: Mueller, G.J. (Ed.) Salmon Saprolegniasis. Report to Bonneville Power Administration. Environment, Fish and Wildlife Division, Portland, Oregon. pp. 33–66. 2) van den Berg, A.H., McLaggan, D., Diéguez-Uribeondo, J. & van West, P. (2013) The impact of the water moulds Saprolegnia diclina and Saprolegnia parasitica on natural ecosystems and the aquaculture industry. Fungal Biology Reviews 27: 33–42. http://dx.doi.org/10.1016/j.fbr.2013.05.001
oomycete_OTU_280	Plectospira	Plant Pathogen	doi.org/10.11646/phytotaxa.307.3.3
oomycete_OTU_41	Saprolegnia	Pathotroph-Saprotroph Oportunistic species	1) Beakes, G.W., Wood, S.E. & Burr, A.W. (1994) Features which characterize Saprolegnia isolates from salmon fish lesions – a review. In: Mueller, G.J. (Ed.) Salmon Saprolegniasis. Report to Bonneville Power Administration. Environment, Fish and Wildlife Division, Portland, Oregon. pp. 33–66. 2) van den Berg, A.H., McLaggan, D., Diéguez-Uribeondo, J. & van West, P. (2013) The impact of the water moulds Saprolegnia diclina and Saprolegnia parasitica on natural ecosystems and the aquaculture industry. Fungal Biology Reviews 27: 33–42. http://dx.doi.org/10.1016/j.fbr.2013.05.001
oomycete_OTU_55	Apodachlya	Soil-Plant Saprotroph	doi.org/10.1007/BF00418292
oomycete_OTU_65	Paralagenidium	Animal Pathogen	Grooters AM, 2008. Pythiosis and Lagenidiosis. In: Boagura JD, Twedt DC (eds), Kirk's Current Veterinary Therapy XIV. Saunders Elsevier, St. Louis, MO, pp. 1268e1271.
oomycete_OTU_79	Lagenidium	Pathotroph-Saprotroph Oportunistic species	doi.org/10.1007/s13225-014-0298-1
oomycete_OTU_85	Saprolegnia	Pathotroph-Saprotroph Oportunistic species	1) Beakes, G.W., Wood, S.E. & Burr, A.W. (1994) Features which characterize Saprolegnia isolates from salmon fish lesions – a review. In: Mueller, G.J. (Ed.) Salmon Saprolegniasis. Report to Bonneville Power Administration. Environment, Fish and Wildlife Division, Portland, Oregon. pp. 33–66. 2) van den Berg, A.H., McLaggan, D., Diéguez-Uribeondo, J. & van West, P. (2013) The impact of the water moulds Saprolegnia diclina and Saprolegnia parasitica on natural ecosystems and the aquaculture industry. Fungal Biology Reviews 27: 33–42. http://dx.doi.org/10.1016/j.fbr.2013.05.001
oomycete_OTU_88	Lagenidium	Pathotroph-Saprotroph Oportunistic species	doi.org/10.1007/s13225-014-0298-1

Table S10 Taxonomy of the 20 most abundant OTUs, resulting form the Core Biome analysis of the fungal and oomycete microbiomes

Abund. Pos.	Feature ID	Taxon
1	oomycete_OTU#1	k__Chromista; p__Oomycota; c__Oomycetes; o__Peronosporales; f__Peronosporaceae; g__Phytophthora; s__Phytophthora plurivora
2	oomycete_OTU#6	k__Chromista; p__Oomycota; c__Oomycetes; o__Pythiales; f__Pythiaceae; g__Pythium; s__Pythium paroecandrum
3	oomycete_OTU#10	k__Chromista; p__Oomycota; c__Oomycetes; o__Peronosporales; f__Peronosporaceae; g__Phytophthora; s__Phytophthora psychrophila
4	oomycete_OTU#3	k__Chromista; p__Oomycota; c__Oomycetes; o__Peronosporales; f__Peronosporaceae; g__Phytophthora quercina
5	oomycete_OTU#9	k__Chromista; p__Oomycota; c__Oomycetes; o__Peronosporales; f__Peronosporaceae; g__Phytophthora; s__Phytophthora citrophthora
6	oomycete_OTU#17	k__Chromista; p__Oomycota; c__Oomycetes; o__Pythiales; f__Pythiaceae; g__Pythium; s__Pythium heterothallicum
7	oomycete_OTU#15	k__Chromista; p__Oomycota; c__Oomycetes; o__Pythiales; f__Pythiaceae; g__Pythium; s__Pythium irregulare
8	oomycete_OTU#7	k__Chromista; p__Oomycota; c__Oomycetes; o__Pythiales; f__Pythiaceae; g__Elongisporangium; s__Elongisporangium undulatum
9	oomycete_OTU#4	k__Chromista; p__Oomycota; c__Oomycetes; o__Peronosporales; f__Peronosporaceae; g__Phytophthora; s__Phytophthora cinnamomi
10	oomycete_OTU#16	k__Chromista; p__Oomycota; c__Oomycetes; o__Pythiales; f__Pythiaceae; g__Pythium
1	fungi_OTU#3	k__Fungi; p__Mucoromycota; c__; o__Mortierellales; f__Mortierellaceae; g__Mortierella; s__Mortierella elongata
2	fungi_OTU#2	k__Fungi; p__Ascomycota; c__Sordariomycetes; o__Hypocreales; f__Nectriaceae; g__Fusarium
3	fungi_OTU#8	k__Fungi; p__Basidiomycota; c__Agaricomycetes; o__Russulales; f__Russulaceae; g__Russula; s__Russula praetervisa
4	fungi_OTU#11	k__Fungi; p__Mucoromycota; c__; o__Mortierellales; f__Mortierellaceae; g__Mortierella; s__Mortierella elongata
5	fungi_OTU#24	k__Fungi; p__Basidiomycota; c__Tremellomycetes; o__Filobasidiales; f__Piskurozymaceae; g__Solicoccozyma
6	fungi_OTU#9	k__Fungi; p__Ascomycota; c__Sordariomycetes; o__Hypocreales; f__Nectriaceae; g__Thelonectria
7	fungi_OTU#25	k__Fungi; p__Ascomycota; c__Eurotiomycetes; o__Eurotiales; f__Aspergillaceae; g__Penicillium
8	fungi_OTU#51	k__Fungi; p__Ascomycota; c__Sordariomycetes; o__Hypocreales; f__Hypocreaceae; g__Trichoderma
9	fungi_OTU#48	k__Fungi; p__Ascomycota; c__Sordariomycetes; o__Hypocreales; f__Bionectriaceae; g__Clonostachys
10	fungi_OTU#15	k__Fungi; p__Ascomycota; c__Sordariomycetes; o__Hypocreales; f__Hypocreaceae; g__Trichoderma
